# QTL mapping and genomic analyses of earliness and fruit ripening traits in a melon recombinant inbred lines population supported by *de novo* assembly of their parental genomes

**DOI:** 10.1093/hr/uhab081

**Published:** 2022-01-19

**Authors:** Elad Oren, Galil Tzuri, Asaf Dafna, Evan R Rees, Baoxing Song, Shiri Freilich, Yonatan Elkind, Tal Isaacson, Arthur A Schaffer, Yaakov Tadmor, Joseph Burger, Edward S Buckler, Amit Gur

**Affiliations:** 1 Plant Science Institute, Agricultural Research Organization, Newe Ya’ar Research Center, P.O. Box 1021, Ramat Yishay 3009500, Israel; 2 The Robert H. Smith Institute of Plant Sciences and Genetics in Agriculture, Faculty of Agriculture, The Hebrew University of Jerusalem, Rehovot, Israel; 3 Plant Breeding and Genetics Section, Cornell University, Ithaca, NY 14853, USA; 4 Plant Science Institute, Agricultural Research Organization, The Volcani Center, P.O. Box 15159, Rishon LeZiyyon 7507101, Israel; 5 United States Department of Agriculture-Agricultural Research Service, Robert W. Holley Center for Agriculture and Health, Ithaca, NY 14853, USA

## Abstract

Earliness and ripening behavior are important attributes of fruits on and off the vine, and affect quality and preference of both growers and consumers. Fruit ripening is a complex physiological process that involves metabolic shifts affecting fruit color, firmness, and aroma production. Melon is a promising model crop for the study of fruit ripening, as the full spectrum of climacteric behavior is represented across the natural variation. Using Recombinant Inbred Lines (RILs) population derived from the parental lines “Dulce” (*reticulatus*, climacteric) and “Tam Dew” (*inodorus*, non-climacteric) that vary in earliness and ripening traits, we mapped QTLs for ethylene emission, fruit firmness and days to flowering and maturity. To further annotate the main QTL intervals and identify candidate genes, we used Oxford Nanopore long-read sequencing in combination with Illumina short-read resequencing, to assemble the parental genomes *de-novo*. In addition to 2.5 million genome-wide SNPs and short InDels detected between the parents, we also highlight here the structural variation between these lines and the reference melon genome. Through systematic multi-layered prioritization process, we identified 18 potential polymorphisms in candidate genes within multi-trait QTLs. The associations of selected SNPs with earliness and ripening traits were further validated across a panel of 177 diverse melon accessions and across a diallel population of 190 F1 hybrids derived from a core subset of 20 diverse parents. The combination of advanced genomic tools with diverse germplasm and targeted mapping populations is demonstrated as a way to leverage forward genetics strategies to dissect complex horticulturally important traits.

## Introduction

Earliness of maturity is an important trait of crop plants with a direct impact on production efficiency and stress tolerance. Horticultural earliness, also referred to as days to harvest (DtH), was previously dissected in tomato to its components – time from sowing to first female flower (flowering time), and number of days for fruit development and ripening [1]. Flowering time has been extensively studied in Arabidopsis and in grasses such as wheat, rice and maize, where it constitutes an important component in earliness, though the genetic architecture differs between self-pollinating and outcrossing plants [2].

In fleshy fruits, fruit development and ripening are considered as the main components determining earliness. Fruit development consists of carpel cells expansion and differentiation, and ripening is a complex process that typically includes modifications in fruit color, texture, composition and profile of sugars, acids, and volatiles [3, 4]. Ripening behavior can be classified as non-climacteric or climacteric, based on the presence or absence of ethylene hormone synthesis and increased respiration at the beginning of ripening [5]. The main factors in climacteric ripening are ethylene biosynthesis and perception. Related genes and mutants are extensively described in Arabidopsis and tomato: ACC synthase (ACS) and ACC oxidase (ACO) [6] are key enzymes in the ethylene pathway, and ethylene perception is mediated by receptors (ETRs) [7]. The ethylene pathway has also been studied in melon [8–12], which is considered a distinctive model for the study of fruit ripening behavior, as the full spectrum of non-climacteric to climacteric behavior is represented across its natural variation [13]. As a result, genotypes may display different combinations of these behaviors as recently documented–aromatic individuals that do not abscise or do not change external color and flesh softening that happens in both climacteric and non-climacteric backgrounds [14, 15]. Populations originating from the non-climacteric *inodorus* group and climacteric (e.g. *cantalupensis* group) lines have enabled QTL mapping of abscission formation [16], ethylene biosynthesis and flesh firmness [17–19], followed by cloning of a ripening related causative gene, *CmNAC-NOR*, an orthologue to the tomato ripening mutant *NOR* gene [20]. Another QTL involved with the onset of climacteric ripening was recently mapped to a 150 Kb interval on chromosome 8 [14]. Comparative transcriptional profiling of climacteric versus non-climacteric accessions identified genes associated with ethylene biosynthesis (*CmACS, CmACO*), cell wall integrity, carotenoid accumulation and sugar metabolism [21]. Various candidate genes associated with softening and sugar buildup have been suggested based on Genome-Wide Association (GWA) analyses performed on diverse melon collections [22, 23].

Flowering initiation is an integrated response to environmental and endogenous cues through a network of pathways responding to factors such as photoperiod, vernalization, aging, autonomous flowering, and gibberellic acid (GA) [24]. Recently described components in the GA pathway, that directly affect flowering time regulation, are WRKY transcription factors, a large gene family also participating in abiotic and biotic stress responses [25, 26]. The genetic factors controlling earliness have been described in tomato [27–29]. In melons, previous studies have identified several QTLs for earliness on chromosomes 1, 2, 9, 10 and 12 [30], and for flowering time on chromosomes 6 and 7 [19].

The genomic resources for melon are constantly improving. Since the first melon reference genome, published in 2012 [31], updated versions have been continuously released [32, 33]. The recent resequencing of 1175 [34] and 297 [35] melon accessions is providing an important resource for characterization of genomic variation, and databases like the Melonet-DB expression atlas [36] and CuGenDB [37] provide broad expression profiles and the latest annotations, pathways and comparative genomics tools. These resources have proved extremely valuable in QTL mapping studies, especially when considering candidate genes [14, 38–40].

Recent advances in long-read sequencing have presented an important addition to the available tools that simplify assemblies and can further elucidate genomic context of QTLs. *De novo* assemblies are becoming more common for model and non-model organisms, and pan-genomes are becoming the new references [41–44]. The study of copy number variations (CNV) and presence-absence variations (PAV), has uncovered extensive genome content variation within tomato, maize and other species [45–47], and demonstrated the major impacts that large SVs can have on fruit flavor, size and yield in tomato [48]. In melon, SVs have been documented as an important source of intra-specific variations [49]. A recent study has characterized in detail small to medium SVs (50 bp – 100Kb) and provided an important layer of information, e.g. annotated PAVs in resistance genes on chromosome 5 [50]. The genome assembly of “Payzawat” melon cultivar using long-read sequencing, detected large inversions across chromosome 6 when compared to the latest version of the melon reference genome [51]. A recent *de novo* assembly of the semi-climacteric “Harukei-3” provides insight to the effect of transposable elements on ripening related gene expression [52].

In the current study, we used a RILs population derived from melon inbred lines differing in their earliness and ripening behavior, to map QTLs related to these traits. Resequencing of parental genomes facilitated detailed genomic analysis of QTL intervals, an expansion of the genomic comparison between our parental lines and an improved QTLs annotation. We also present *de novo* assemblies of their genomes and highlight the structural variations between them, some of which are in context of the detected QTLs. Associations of selected candidate genes and polymorphisms within them were validated across a diverse collection and a large diallele population.

## Results

### Phenotypic variability of earliness, ethylene emission, and fruit firmness across TAD×DUL RILs

**Figure 1:**
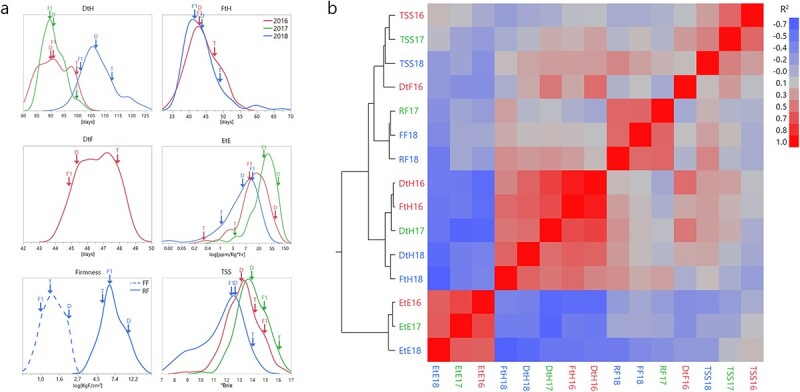
Variation in earliness and ripening traits in the TADxDUL RILs. a) Frequency distributions on entry mean basis over 3 years. Arrows mark the parental (D = “Dulce”, T = “Tam Dew”) and F1 hybrid values. b) Correlation matrix of hierarchically clustered traits that were measured across the experiments. Traits are color coded according to year.

The TAD×DUL RILs population was analyzed over three years for earliness and ripening related traits. We characterize agronomic earliness as days from transplanting to harvest (DtH) and further break it down to its components – days to flower (DtF) and fruit development time (flower to harvest, FtH). A total of 3963 fruits were sampled for DtH across the different experiments, averaging 10 fruits per line per year. Variation in DtH, analyzed on line-mean basis, is substantial and distributes in a transgressive manner across nearly 20 days (83–101 days, Table 1**,**Fig. 1a). In the open field (OF) trials, “Dulce” and the F1 matured after 90 days, while “Tam Dew” ripened after 100 days. Nearly a third of the population matured either earlier or later than the parents. In the net house (NH) experiment, the ripening process was slower by 10 days on average (93–125 days), the F1 matured a week before “Dulce” and the difference between the parental lines was reduced to four days with a distribution similar to the open field (Fig. 1a). DtF was measured across the population by tagging all visible female flowers at anthesis and collecting the tags date from all fruits during harvest (Supplementary Fig. 3a and b). FtH was calculated for each fruit as the time from anthesis to harvest, and this trait was evaluated also in the net house, where days from manual pollination were tracked. When considering these components, FtH accounts for most of the variation in earliness, and ranges between 34 and 55 days, while the variation in DtF is less than a week (44–49 days). Transgressive segregation is also displayed in FtH variation with RILs in the population having shorter fruit development time than “Dulce” or longer than “Tam Dew”. In both, the earlier parent displays a slight dominance (Table 1**,**Fig. 1a). FtH and DtH values were moderately correlated between both open field and net house experiments (*r* = 0.55–0.6, Fig. 1b) with FtH displaying nearly identical distributions between environments (Fig. 1a). Both FtH and DtH were found to be highly heritable in both environments (*h*^2^ = 0.56–0.72, Table 1). Heritability calculated for DtF was slightly lower, *h*^2^ = 0.34.

**Table 1:** Description, abbreviation, and genetic properties of earliness and ripenning traits in the TAD × DUL RILs

**Trait name**	**Abbr.**	**Units**	**Description**	**Open Field /Net house**	**Mean**	**Range**	**h** ^**2**^	**a** ^**a**^	**d** ^**b**^	**d/a**
Days to harvest	DtH	days	Days from sowing to harvest	OF	91 ± 4.5	83 - 101	0.72	4.4	-5.2	-1.2
				NH	108 ± 6.1	93 - 125	0.56	2.2	-9.1	-4.1
Days to flower	DtF	days	Days from sowing to anthesis	OF	47 ± 1.2	44 - 49	0.34	1.3	-1.8	-1.4
				NH	-	-	-	-	-	-
Fruit development time	FtH	days	days from anthesis to flowering	OF	43.9 ± 4.4	34 - 55	0.67	1.9	-1.8	-0.9
				NH	43.5 ± 5.0	36 - 67	0.62	3.3	-1.3	-0.4
Ethylene emission	EtE	μL kg^−1^ h^−1^	fruit ethylene production at maturity	OF	31.4 ± 21.1	0.5 - 115.0	0.58	41	-23	-0.6
				NH	8.9 ± 7.6	0.01 - 34.9	0.7	16	-5.1	-0.3
Rind firmness	RF	KgF cm^-2^	Rind firmness	OF	17 ± 5.6	3.9 - 26.0	0.72	-	-	-
				NH	7.3 ± 2.5	3.3 - 16.7	0.66	2.8	-2	-0.7
Flesh firmness	FF	KgF cm^-2^	Flesh firmness	OF	-	-	-	-	-	-
				NH	1.4 ± 0.4	0.6 - 2.4	0.62	0.5	-0.9	-1.8

a Additive value, calculated as |TAD-DUL|/2

b Dominance values calculated as the deviation of F1 (TADxDUL) from mid parent value.

Ethylene emission (EtE) of the RILs, parental lines and their F1 hybrid was limited to two fruits from separate plants per replicate. A total of 1258 fruits were sampled in the open field experiments and 536 fruits from the net house, averaging 11 fruits per line. This trait was also found to be highly heritable (*h*^2^ = 0.60–0.70, Table 1) and demonstrated high correlations between environments (*r* = 0.60–0.72, Fig. 1b). The distribution observed for EtE is of a logarithmic nature, with “Dulce”, the climacteric parent, producing 85 μL kg^−1^ h^−1^ and “Tam Dew”, the non-climacteric line, producing an average of 1.6 μL kg^−1^ h^−1^ in the open field experiments (Fig. 1a). The F1 produced around 20 μL kg^−1^ h^−1^, in absolute values, which in essence reflects an additive mode of inheritance due to the logarithmic nature of this trait (Fig. 1a, log(d/a) = 0.3 and 0.4 in the open field and net house, respectively). EtE levels measured across the population in the field experiment range between 0.5–115 μL kg^−1^ h^−1^, with most of the RILs within the parents’ range, except for several RILs that show transgressive segregation on both sides. The same pattern was visible in the net house, though overall ethylene emission values are lower in this experiment (Fig. 1a).

Rind firmness (RF) was evaluated in one open field experiment (2017) and in the net house experiment (2018), while flesh firmness (FF) was only evaluated in the net house experiment. There is moderate positive correlation between the open field and the net house (*r* = 0.57) with RF values in the open field between 3–26 kg cm ^−2^ and in the net house 3–17 kg cm ^−2^. FF values range between 0.6 and 2.7 kg cm ^−2^, with “Dulce” about twice as firm compared to “Tam Dew” in both tissues. RF values display a much wider range than FF (Table 1), but both traits are of a logarithmic nature and when analyzed as such they are similar in range and distribution (Fig. 1a) and positively correlated (***r* = 0.56,**Fig. 1b**)**. Both traits are characterized by transgressive segregation across the population, with approximately a third of the RILs softer or harder than the parents. In the net house, both RF and FF display dominant inheritance with the F1 fruits not significantly different from “Tam Dew” (RF d/a = −0.7, FF d/a = −1.8, Table 1**,**Fig. 1a).

Sugar content (total soluble solids - TSS) was measured on 3510 mature fruits across all experiments with a mean of 8 fruits per line in the open field experiments and 4 fruits per line in the net house. Interestingly, while both parents have high TSS, with “Tam Dew” constantly a couple of degrees brix sweeter than “Dulce” (~15 vs 13°brix), substantial transgressive segregation is observed across the RILs (9.4–16.4°brix). The environmental effects and G × E interactions in this trait are apparent, as distributions are moderately correlated between the open field experiments but not so between the open field and the net house, where TSS values are lower (Fig. 1a and 1b). TSS displays the lowest heritability of all traits, *h*^2^ = 0.33 in the open field and 0.58 in the net house (Table 1).

The full matrix of correlations between traits and years (Fig. 1b) reflect the expected clustering of traits to physiological groups. For example, fruit firmness traits—RF and FF—are positively correlated, and so are DtH and FtH that are related to earliness. This analysis also emphasizes the inherent negative correlations between ripening behavior (e.g. EtE) and earliness traits. The correlation between DtF and EtE was −0.3 (*p* = 0.0004). A stronger negative correlation with EtE is observed for both FtH and DtH in the open field, ranging between *r* = −0.60 and − 0.65. This negative relation is even more pronounced in the net house (*r =* −0.61 for DtH and EtE, and r = −0.69 for FtH and EtE). Interestingly, this analysis also shows that ripe fruit TSS is not correlated with ripening behavior or with earliness traits (Fig. 1b), as also shown in a previous study [53].

### QTL mapping

QTL mapping is performed as previously discussed [39], using a combination of methods, including stepwise and composite interval mapping. QTLs that are significant in at least two experiments are considered robust and two-way epistatic interactions were tested among these QTLs. QTL models for each trait are constructed based only on robust QTLs and are tested on each experiment separately.

### QTLs for earliness and ethylene emission

**Figure 2:**
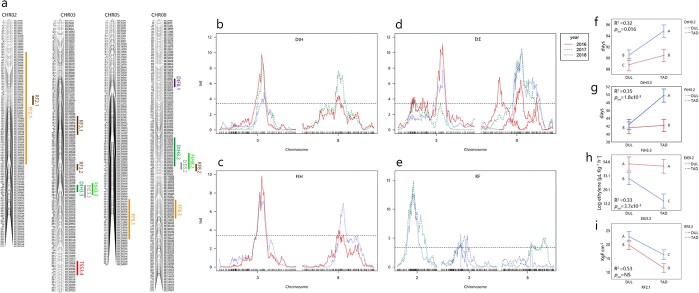
QTLs for earliness and ripening traits in the TADxDUL RILs. a) Linkage maps of chromosomes with robust QTLs mapped across three years in this study. b-e) LOD score plots for the major QTLs. Dashed horizontal lines are significance threshold. b) Days to Harvest (DtH). c) Flowering to Harvest (FtH). d) Ethylene Emission (EtE). e) Rind Firmness (RF). f-i) Interaction plots between major QTLs. statistically different means designated by different letters.

DtH, FtH and EtE are all phenotypically correlated in our population across the different experiments (Fig. 1b), and this is evident also by the co-localization of the two main QTLs for these traits. On chromosome 3, they all share an overlapping physical interval of ~300 Kb. *FtH3.3* and *EtE3.3* have a genetic interval of 9 cM and *DtH3.3* is slightly smaller – 4 cM (Fig. 2a). This multi-trait QTL is consistent across all experiments (Fig. 2b–d), and accounts for 24% of the genetic variation in DtH and FtH, and 18% in EtE (Table 2). The “Tam Dew” allelic effect in *FtH3.3* delays ripening by 2.2 days on average and this allele in *EtE3.3* is associated with decrease of 6.3 μL kg^−1^ h^−1^ in ethylene emission. On chromosome 8, *FtH8.2* and *EtE8.2* share the same peak, but the genetic and physical confidence intervals for *FtH8.2* are double the size of *EtE8.2* (12 vs 6 cM and 440 vs 250 Kb, respectively, Fig. 2c, d**,**Table 2). *FtH8.2* accounts for 15% of the genetic variation and *EtE8.2* accounts for 13%. QTL *DtH8.2* in this common interval accounts for 24% of the genetic variation and was only significant in the open field experiments. *DtH8.2* interval is 21 cM and 2 Mb, and partially overlaps with *FtH8.2* and *EtE8.2*. On *FtH8.2* the “Tam Dew” allelic effect delays ripening by 2 days on average and *EtE8.2* mitigates ethylene production by 6 to 14 μL kg^−1^ h^−1^ (Table 2). When integrating the effects of the multi-trait QTLs on chromosomes 3 and 8 into a model fitted for DtH and FtH, they have an additive effect of 6–8 days and account for ~30% of the genetic variation (Supplementary Fig. 4**a–e**). Significant epistatic interaction between *FtH3.3* and *FtH8.2* was detected only in the net house (*p* = 1.8x10^−5^**,**Supplementary Fig. 4e), and likewise in DtH for the net house and one of the open field experiments (Supplementary Fig. 4b and c). Another epistatic interaction between both loci is also visible for EtE, significant only in the open field experiments (*p* = 0.013 and *p* = 0.0037, Supplementary Fig. 4f–h). Overall, the combined effect of the QTLs for EtE, FtH, and DtH in these two loci—3.3 and 8.2—is not different from additive performance and a two loci model for EtE reflect three distinct levels of ethylene production and account for 33% of the genetic variation (Fig. 2h). DtF, the first component of DtH, has one significant QTL, *DtF8.1*, on a separate region of chromosome 8, at 4.25 Mb. This QTL accounts for 18% of the genetic variation and spans 400 Kb and 8 cM on the linkage map (Table 2).

**Table 2:** Robust QTLs for earliness and ripening related traits in the TAD *×* DUL RILs by composite and stepwise interval mapping

**Trait**	**QTL** ^**a**^ **name**	**Chr**	**LOD** ^**b**^	**Genetic QTL peak position (cM)**	**Genetic QTL confidence interval (cM)** ^d^	**Physical QTL position (Mb)**	**Physical QTL confidence interval (Mb)** ^e^	**% Var explained** ^f^	**Additive effect** ^g^
DtH	DtH3.3	3	9.8	163.7	4.1	23.84	23.84–24.09	23.9	−2.75
DtH	DtH8.2	8	7.6	139.8	20.9	7.10	6.96–8.62	17.6	−1.72
DtF	DtF8.1	8	6.5	71.5	7.9	4.25	4.10–4.53	18.2	−0.5
FtH	FtH3.3	3	9.8	163.7	8.9	23.84	23.80–24.10	24.5	−2.23
FtH	FtH8.2	8	6.9	158	12.1	8.64	8.35–8.79	15.6	−2.01
EtE	EtE3.3	3	11.1	172.4	8.7	24.35	23.84–24.35	18.2	6.3^b^
EtE	EtE8.2	8	10.5	158	6.4	8.64	8.54–8.79	13.1	14.5^b^
RF	RF2.1	2	14.9	85.5	5.1	6.36	5.86–6.54	27.8	3.10
RF	RF3.1	3	5.8	102.5	15.3	14.69	14.42–14.70	9.9	0.94
RF	RF3.2	3	5.5	138.6	5.7	22.70	22.03–22.70	9.3	−0.93
RF	RF8.2	8	4.2	158.0	7.6	8.64	8.55–8.90	10.4	−1.00
FF	FF8.3	8	6.3	194.9	19.3	25.64	24.34–27.04	14.3	−0.16
FF	FF5.1	5	4.2	151.8	32.6	26.59	26.44–27.95	9.1	−0.12
FF	FF2.1	2	4.0	82.3	93.3	5.34	1.90–17.54	8.8	0.12

a QTL names are composed of trait abbreviation, chromosome number and QTL number

b Numbers are non-standardized values (logarithmic transformation was applied for mapping)

c Maximum LOD score for consensus QTLs. Main effects from R/qtl scanone and secondary from stepwise analysis

d Two neutral loci involved in epistatic interaction

e Interval based on at least 1.5 LOD score drop

f Interval bases on flanking markers physical position

g Maximum R square for each QTL

h Positive additive effect when DUL alleles contribute to trait score and negative for TAD alleles

i Two-way ANOVA using peak QTL marker and year as factors. p – values: ^*^ < 0.05; ^**^ < 0.01; ^**^* < 0.001

### QTLs for rind and flesh firmness

Fruit firmness was measured separately for rind and flesh, which are moderately correlated (Fig. 1b and materials and methods). This is also apparent in the QTL analysis, which yielded a shared major QTL for both tissues (*RF2.1* and *FF2.1*, Fig. 2a), while the rest of the QTLs for these traits did not overlap. Four QTLs are mapped for RF, on chromosomes 2, 3 and 8, with the main being *RF2.1*, accounting for 28% of the genetic variation with an interval size of 5.1 cM and 700 Kb. “Dulce” allele at this QTL is associated with increased firmness by 3.1 KgF cm^−2^. On chromosome 3, *RF3.1* is 15 cM long, but the physical size of this interval is difficult to estimate due to genomic rearrangements in this region, that are discussed in more details in the structural variation section. *RF3.2* is 5 cM long and spans across 670Kb, and *RF8.2,* on chromosome 8, is 7.6 cM across 450 Kb. Each of these secondary RF QTLs accounts for ~10% of the genetic variation with an additive effect of about 1 KgF cm^−2^ (Table 2). A model composed of *RF2.1* and *RF8.2* accounts for 35–53% of the variation and can distinguish between four distinct levels of RF in the open field experiment (Fig. 2i) and three in the net house (Supplementary Fig. 4j). FF analysis yielded three QTLs on chromosomes 2, 5 and 8. *FF8.3*, the main QTL for this trait, 19 cM long and covers 670 Kb, accounts for 14% of the genetic variation. *FF5.1* is 33 cM long across 1.5 Mb and accounts for 9% of the genetic variation. *FF2.1* practically spans half of the chromosome, including the interval of *RF2.1*. Since FF was only measured in the net house experiment (2018), to support the validity of QTLs for this trait, we analyzed the correlation between the five replications in the net house experiment. All correlations were significant and above *r* = 0.55 (Supplementary Fig. 5a), justifying a unified QTL analysis of all blocks (Supplementary Fig. 5b). A fitted model including the two major QTLs, *FF5.1* and *FF8.3* can significantly distinguish between three levels of flesh firmness (1.16–1.74 KgF cm^−2^) and account for a third of the total genetic variation in this trait (Supplementary Fig. 4k).

A total of 31 QTLs were detected across the earliness and ripening-related traits (Supplementary Table 1). Fourteen robust QTLs on chromosomes 2, 3, 5 and 8, are considered major contributors to earliness and ripening related traits (Table 2). Two loci, on chromosomes 3 and 8 can be described as major, multi-trait QTLs, as they contain seven of the robust QTLs (Fig. 2a).

### Annotation of QTL intervals and prioritization of candidate genes

**Figure 3:**
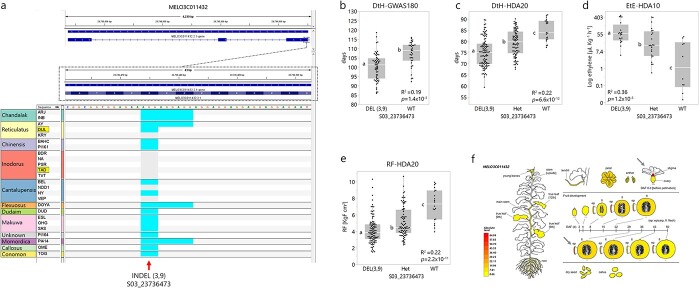
Characterization of *MELO3C011432*-WRKY family transcription factor a) InDels (3 bp or 9 bp) in the first exon, across 20 diverse accessions from the core panel. Colors according to horticultural group. Tam Dew and “Dulce” highlighted. b-e) Association of the InDel with different traits. Statistically different means designated by different letters. b) Days to Harvest (DtH) across 100 melon accessions from the diverse collection. c) DtH across *HDA20* population. d) Ethylene Emission (EtE) across *HDA10* population. e) Rind Firmness (RF) across the *HDA20* population. f) Spatial expression profile of *MELO3C011432* as presented in MelonetDB [36]. Arrows mark tissues with high expression levels.

To extract further downstream information from QTL mapping results, we designed and implemented a systematic workflow to assist in the integration of multiple data-layers. This process facilitates effective annotation and prioritization of candidate genes within QTL genomic intervals, using a combined score matrix (Supplementary Fig. 2). Permissive confidence intervals of 2 LOD scores around QTL peaks were used as targets for QTL annotation. Five layers of information are included in the prioritization process: 1) Score for proximity of each gene to QTL peak. 2) Annotation and description of gene models – score is based on predicted gene function and relevancy to the target trait. 3) Spatial and temporal expression profiles of genes – score is based on alignment of expression profile (through development and plant organs from MelonetDB [36]) with the target trait, and comparative expression analysis between parental lines. 4) Annotations of genomic polymorphisms between parental lines: We started this process with a comprehensive set of 2 493 544 SNPs extracted from the resequenced parental lines, “Tam Dew” and “Dulce”. These polymorphisms were aligned to the latest version of the reference-genome-based gene models (CM4.0) [32] and annotated for their predicted effects, using the SnpEff software [54]. Following removal of intergenic regions (excluding UTR ranges up and downstream of predicted genes), a set of 226 281 annotated SNPs were used for further analyses where each SNP was ranked based on its predicted impact. 5) Association of candidate SNPs across additional multi-allelic populations – score is based on the significance of the SNP association in our GWAS panel and diverse half-diallel populations. The half-diallele populations are derived from our core subset of re-sequenced parental lines, and as such facilitated analysis of association of earliness and ripening behavior traits, that were collected on these populations, against any candidate polymorphism. These multi-layered descriptions are integrated into an indexed general score for each candidate gene (Supplementary Fig. 2). This analysis that included 733 genes across all the robust QTLs that were mapped in the current study, resulted in a set of 18 high priority candidates that are presented in Supplementary Table 2 – five related to earliness, 5 to ethylene emission and 11 to rind and flesh firmness. We elaborate on three prominent earliness and ripening behavior candidates:

***MELO3C011432*.** In the multi-trait QTL on chromosome 3 (*FtH3.3*, *DtH3.3* and *EtE3.3*), out of 41 genes annotated across the confidence interval, *MELO3C011432*, a WRKY family transcription factor, received a high score, with a codon deletion in “Dulce” (3 bp InDel in the first exon, Fig. 3a). This gene which is associated with developmental processes, e.g. response to biotic and abiotic stresses, ethylene, senescence, seed germination, and flowering time, seems to be expressed mainly in the stigma and rind (Fig. 3f). Another important supportive information for this gene as candidate is the significant associations found with DtH, EtE and RF across our GWAS panel and diallel populations (*HDA10* and *HDA20*, tested in three different field experiments, Fig. 3b–e). These diverse populations exposed that this InDel is an SSR-type polymorphism (3 or 9 bp deletions), where both deleted alleles are associated with similar phenotypic effects compared to the reference genotype. In the GWAS panel, the deletion alleles (3 bp and 9 bp, combined) were associated with significant earlier ripening by 10 days (Fig. 3b*, p* = 1.4x10^−5^). In the *HDA20* population, similar allelic effect on DtH is shown with a clear additive mode of inheritance, where heterozygotes are intermediate to the homozygote genotypes (Fig. 3c*, p* = 6.6x10^−12^). The effect of this locus on EtE was validated in the *HDA10* population where the deletion alleles are associated with increased ethylene production by nearly 60 μL kg^−1^ h^−1^, with additive mode of inheritance (Fig. 3d*, p* = 1.2x10^−5^). Significant association of this gene with fruit firmness was shown also across the *HDA20* population, where the deletion alleles were softer in 3 Kg cm^−2^ than wild-type (reference allele) and heterozygotes are intermediate to both homozygotes (Fig. 3e*, p* = 2.2x10^−11^).

**Figure 4:**
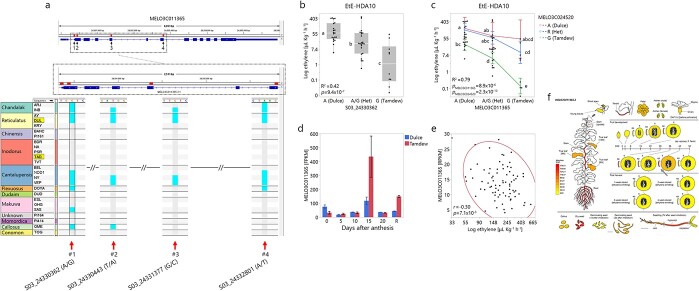
Characterization of *MELO3C011365*-Transducin/WD40 repeat-like superfamily protein. a) Four SNPs in *MELO3C11365* across 20 diverse accessions of the core panel. Colors according to horticultural group. “Tam Dew” and “Dulce” highlighted. SNP#1–splice site position; SNPs#2,3–missense mutations; SNP#4–nonsense mutation. b) Association of SNP#1 with EtE in *HDA10* population. Statistically different means designated by different letters. c) Interaction plot for EtE of *MELO3C011365* and *MELO3C024520* (*ETE8.2)* in *HDA10* population d) Expression profile of *MELO3C011365* from “Tam Dew” and “Dulce” rind across fruit development. R = ripe. e) Correlation between Ethylene emission and *MELO3C011365* expression in ripe fruit across the PI414xDUL RILs population (raw data analyzed from [55]). f) Spatial expression profile of *MELO3C011365* as presented in MelonetDB [36].

**MELO3C011365**. Another candidate gene in *EtE3.3* QTL is *MELO3C011365*, a transducin/WD40 repeat-like superfamily protein, described as a large family of proteins involved in signal transduction and coordinating protein–protein interactions. Forty-eight genes are annotated within *EtE3.3* and *MELO3C011365* is located 20 Kb from the QTL peak. We detected several high impact polymorphisms in this gene, including a nonsense mutation, leading to a premature stop codon, two missense mutations, and a splice site region SNP (Fig. 4a). SNP S03_24330362 showed the strongest association with our EtE data from the *HDA10* population with 55 μL kg^−1^ h^−1^ difference between homozygote allelic groups and intermediate performance of heterozygotes (Fig. 4b, *p* = 9.4x10^−7^). To test the combined effects of *EtE3.3* and *EtE8.2* across our diallel population, we analyzed *MELO3C011365* with *MELO3C24520*–a recently suggested EtE candidate located within *EtE8.2* [14]. Jointly, in a two-way ANOVA, these QTLs explained 79% of the variation across the *HDA10* population, with a difference of 120 μL kg^−1^ h^−1^ between the combination of contrasting alleles at both loci (Fig. 4c). Further supporting *MELO3C011365* as a candidate is the differential expression measured in rind tissues from both parents, where “Tam Dew” displays significantly higher values at 15 days after anthesis (DAA) and in ripe fruits (Fig. 4d). Another layer of evidence is provided by the negative correlation calculated between the expression of *MELO3C011365* and EtE values that were measured in parallel from ripe flesh samples in the “PI414”x“Dulce” RILs population (Fig. 4e). According to MelonetDB [36], this gene is expressed in root, shoot and a peak in fruit rind at 45 DAA (Fig. 4f).

**Figure 5:**
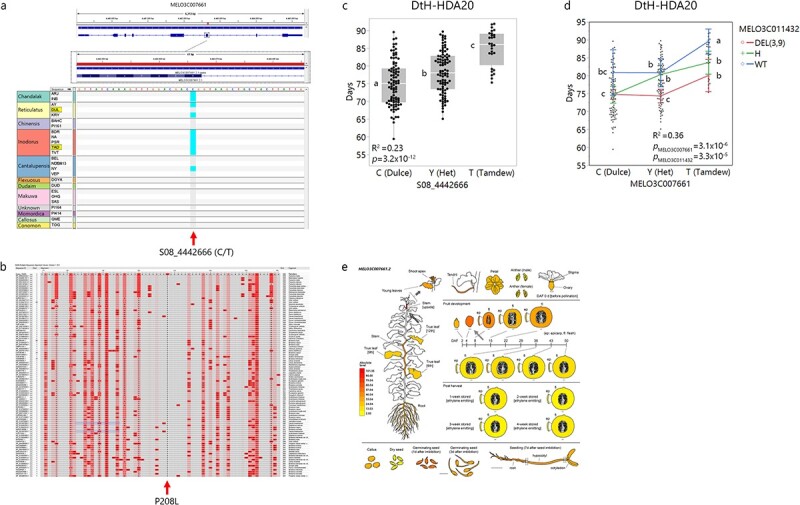
Characterization of *MELO3C007661*-Transmembrane protein, putative. a) Non-synonymous SNP in exon 5 of *MELO3C007661* across 20 diverse accessions of the core panel. Colors according to horticultural group. “Tam Dew” and “Dulce” highlighted. b) *MELO3C007661* protein sequence alignment across 101 plant species from NCBI COBALT multiple sequence alignment viewer (Papadopoulos and Agarwala 2007). The conserved Tam Dew’s proline to leucine substitution caused by the SNP in exon 5 is marked by red arrow (P208L). c) Association of exon5 SNP with DtH in *HDA20* population. Statistically different means designated by letters. d) Interaction plot for the effects of *MELO3C007661* (*DtF8.2*) and *MELO3C011432* (*DtH3.3)* on Days to Harvest (DtH) across *HDA20* population. e) Spatial expression profile of *MELO3C011365* as presented in MelonetDB [36]. Arrows mark tissues with high expression levels.

***MELO3C007661*.** In *DtF8.1*, the major flowering time QTL, out of 48 possible genes within the confidence interval, *MELO3C007661*, a transmembrane protein putative gene, located 190 Kb from the QTL peak, was ranked high as a possible candidate gene with one substantial mutation causing an amino acid (AA) substitution in exon 5 (SNP S08_4442666, Fig. 5a). This projected AA change in “Tam Dew” is a proline to leucine substitution, in a site that appears to be conserved when comparing this protein sequence across multiple plant species (P208L, Fig. 5b). Proline is a neutral and cyclic amino acid, while leucine is hydrophobic, and this substitution was categorized as affecting protein function by both SIFT and PROVEAN based on comparisons to 33 and 54 protein sequences, respectively. DtF was not measured on the diallele population, instead, we used DtH data, which is positively correlated with DtF (*r* = 0.54) and shares a minor QTL with *DtF8.2* (LOD = 2.5, data not shown), to test the association of this polymorphism. Significant association was found between SNP S08_4442666 at *MELO3C007661* and DtH across the multi-allelic *HDA20* population (R^2^ = 0.23, *p* = 3.2 × 10^−12^, Fig. 5c). The difference in DtH between the homozygote allelic groups was 10 days, with heterozygote genotypes being intermediate. To test cumulative earliness effects of QTLs for the components of DtH–DtF and FtH, a combined model of *DtF8.2* (*MELO3C007661*) with the *FtH3.3* candidate, *MELO3C011432*, was tested and significantly accounted for 36% of the genetic variation in DtH (Fig. 5d). The difference in harvest date between contrasting homozygote allelic combinations from both loci was ~14 days (Fig. 5d). This gene is most highly expressed in the plants stem and in fruits 4 DAA (Fig. 5e).

### De novo assembly of “Tam Dew” and “Dulce” genomes and characterization of structural variation

#### Sequencing and genomes assembly

**Figure 6:**
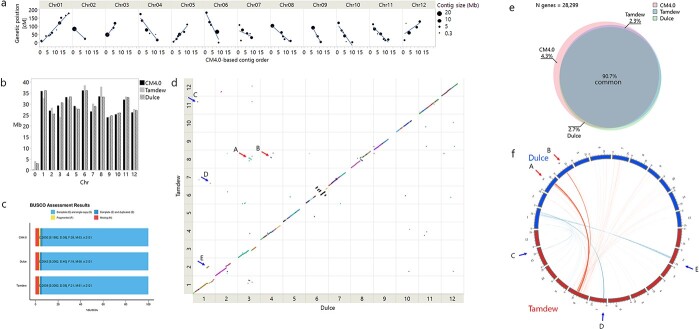
*De novo* assembly and structural variation between the parental genomes. a) *De-novo* contig mapping on TAD×DUL RILs linkage map. Contig orders on each scaffold are presented as rank and correlated with their respective position on the linkage map. Contig size is represented by the size of the marker. Manually corrected locations are marked by grey arrow. b) Comparison of chromosome lengths between CM4.0, “Tam Dew” and “Dulce” assemblies. c) BUSCO assessment of assemblies with respect to gene content and completeness between CM4.0, “Tam Dew” and “Dulce”. d) Whole genome alignment based on unique anchors between genomes. Each dot represents a uniquely aligned feature. Dots are color-coded based on assembly contigs. Arrows point to corresponding translocations that are marked using the same letters on circos plot. e) Circos plot illustrating re-localized genes from Tam Dew’s chromosome 8 to their respective positions on Dulce genome in red, and vice versa from Dulce’s chromosome 1 in blue. f) Venn diagram of gene content comparison. The percentages noted for Tam Dew or Dulce relate to genes missing from the former or latter but shared with CM4.0.

To improve the genomic resources available for QTL annotation, we developed and implemented a bioinformatic workflow integrating both second and third generation sequencing technologies, as illustrated in supplementary Fig. 6, to *de novo* assemble the parental genomes of the RILs population. We generated 15.7 Gb of Oxford Nanopore Technology (ONT) reads of “Tam Dew” and 23.3 Gb of “Dulce”, representing ~43× and ~ 64× coverage of the estimated 400 Mb melon genome, respectively. N_50_ for ONT read lengths was 16.3Kb and 20.2Kb for “Tam Dew” and “Dulce”, respectively. The initial assembly of “Tam Dew” was comprised of 386 contigs with an N_50_ of 3.4 Mb and “Dulce” assembly was comprised of 190 contigs with an N_50_ of 7 Mb (Supplementary Table 3). The contigs passed three rounds of polishing using the ONT reads and three rounds using previously generated illumina short read data (~40× coverage per genome, [56, 57]). After polishing, the order and orientation of contigs were based on the latest melon assembly (DHL92 CM4.0) [32] via reference guided scaffolding, resulting in chromosome-scale pseudomolecules. The scaffolding process was independently validated using unique anchor sequences from each contig that were genetically mapped onto the TAD×DUL RILs linkage map (Fig. 6a). Final genome size was 367 Mb for “Tam Dew” and 365 Mb for “Dulce”, and unmapped sequences in both genomes were less than 4 Mb. Detailed comparisons of chromosome lengths reveal that “Dulce” and DHL92 (CM4.0) chromosomes are mostly similar in size, and on average the differences are of ~500 Kb, except for chromosome 7 where Dulce is shorter by 2.3 Mb (Fig. 6b). Between “Tam Dew” and “Dulce”, however, there are some notable differences on chromosomes 1, 3 and 8, where lengths vary by as much as 6.4 Mb. Completeness of the assemblies, with respect to gene content, showed that approximately 96% of the BUSCO genes were complete and less than 1% fragmented (Fig. 6c). These results are comparable to the latest published melon reference genome [32] indicating that our assemblies contain most of the gene content.

#### Genome annotation

Repetitive elements were annotated using a combination of *de-novo* and homology-based prediction with RepeatModeler2 [59]. After filtering for protein coding sequences, transposable elements were present in 37.9% of “Tam Dew” and 34.8% of “Dulce” genomes, compared to 45.2% of CM4.0 assembly (Supplementary Table 4). Of the identified long terminal repeats (LTRs) – Copia and Gypsy elements were the dominant class, representing 8.7% and 9.4% in “Tam Dew” and “Dulce” genomes, respectively. Gene model annotations were lifted over from the melon reference CDS CM4.0 [32] using a combined strategy of two tools: The first using Liftoff [59], that is based on sequence coverage and identity of aligned exons within each gene. The second was with GEAN [60], that is based on alignment of primary reference CDS to the target genome. After the lift-over, GEAN also validates predicted CDS completeness in the target genome, based on several parameters (start and end codons, conservation of splice sites, ORF structure and no premature stop codons). We have generally found that GEAN is much more stringent but can successfully account for structural variations that potentially impede gene function, where Liftoff might miss the erroneous annotation. For general genome annotation purposes, we relied on the Liftoff set but when studying QTL intervals, we compared the list with GEAN’s results and manually curated differences between the two sets. Liftoff successfully annotated 26 331 genes in “Tam Dew” and 26 423 in “Dulce” out of 28 299 annotated gene models from the reference genome. 25 671 were present in both parental lines, 1216 were unique to CM4.0, 660 were missing in “Dulce” and 752 were missing in “Tam Dew” (Fig. 6e**,**Supplementary Tables 5 and 6).

#### Structural variation (SV)

Using “assemblytics” [61] we characterized the following different SVs – Insertions, deletions, repeats expansions and contractions (differentiating between tandem and repetitive elements) and categorized them according to their sizes – the largest being 50–100 Kb. Overall, we identified 10 740 structural variants in “Tam Dew”, compared to the reference genome, encompassing 40 Mb. The majority (70%) of these were within repetitive elements – 50 of them larger than 50 Kb. 21% were InDels – 6 larger than 50 Kb. In “Dulce”, we identified 11 800 structural variants encompassing 43 Mb, with 69% within repetitive elements – 55 variants larger than 50 Kb. 24% InDels – 7 of these larger than 50 Kb (Supplementary Table 7).

Based on unique anchor sequences identified by the assemblytics algorithm (>10Kb), we manually scanned for inversions and translocations. To increase the confidence in the reported events, we only considered segments with at least two anchor sequences present. Between “Tam Dew” and the reference genome we identified 42 events in total, 12 inversions of which 3 were larger than 1 Mb, and 18 translocations between chromosomes, 4 larger than 1 Mb – the largest being 3.6 Mb from chromosome 1 in the reference to chromosome 2 in “Tam Dew”. In Dulce we identified 32 events in total, 17 inversions of which one was larger than 1 Mb, and 6 translocations between chromosomes, none larger than 1 Mb. Interestingly, the most substantial SV that we detected was on chromosome 6 where we report five large translocation events encompassing nearly half the chromosome (Supplementary Fig. 6**,**Supplementary Tables 8 and 9).

A direct comparison between “Tam Dew” and “Dulce” (using “Dulce” as the reference), yielded 7973 structural variants encompassing 27 Mb. Here too, the majority (66%) were within repetitive elements, 7% larger than 50 Kb, and 28% were InDels, the largest between 10 Kb and 50 Kb, altogether encompassing approximately 2 Mb (Fig. 6d**,**Supplementary Tables 10 and 11). We identified nine inversions between the parental genomes, the largest being a 3 Mb inversion on chromosome 8. Translocations were more abundant – 15 between chromosomes, four larger than 1 Mb with two of these between chromosome 3, 1 and 8 – a validation for these rearrangements is reflected on the independently generated linkage maps based on the RILs population, using each of the parental genomes as a reference (Supplementary Fig. 7**,**Supplementary Table 12). As found in the comparison between “Dulce” and the reference, the major SV on chromosome 6 is also apparent between our parental lines, with five large translocations, spanning nearly 17 Mb, practically half of the chromosome (Fig. 6d**,**Supplementary Fig. 8).

We further analyzed how the structural variation between “Tam Dew” and “Dulce” affected genome-wide gene distribution, and we report that 1119 genes common to both parents (96% single-copy), were re-localized to different chromosomes. 305 genes from chromosome 8 of “Tam Dew” are located on different chromosomes of “Dulce”, mainly on chromosomes 3 and 4. 292 genes from Dulce’s chromosome 1 were mainly translocated to chromosomes 2 and 7 of Tam Dew (Fig. 6f). These results further support the translocations that we report through whole-genome alignments (Fig. 6d**,**Supplementary Fig. 7), as here they are detected with a partially independent gene lift-over approach, based only on exon alignment.

## Discussion

### Transgressive segregation of earliness and ripening traits in the TAD×DUL RILs

Melon is considered an important model crop for studying fruit ripening, as it encompasses the complete spectrum between non-climacteric and climacteric physiologies within the genus, thus enabling the study of natural quantitative variation in ripening behavior [13, 14, 62]. Mapping populations in these studies were derived from crosses between non-climacteric (*inodorus* type) melon, and climacteric types (e.g. *cantalupensis, chinensis* or *reticulatus*). In the current study, we used a RILs population originating from a cross between the *inodorus* line, “Tam Dew” (a Honey Dew variety) and the climacteric line, “Dulce” (*reticulatus* type). A comparison between EtE from our RILs to a recent study using RILs derived from “Piel de Sapo” (*inodorus)* and a *cantalupensis* variety, “Vedrantais” [14], highlights that their EtE ranges were double those measured in our population (0.5–115 μL kg^−1^ h^−1^, compared to 0–286 μL kg^−1^ h^−1^) a difference that may be attributed to the fact that “Vedrantais” is much more climacteric than “Dulce” (225 vs 90 μL kg^−1^ h^−1^). Nevertheless, several common genetic loci related to ripening were mapped in both populations. Earliness and ripening related traits displayed transgressive segregation across our population, as RILs surpassed the parental range (Fig. 1a). A similar transgressive pattern was reported in the IL population originating from the “Vedrantais” (*cantalupensis)* and Makuwa (*agrestis*) parents [19]. Transgressive segregation is typical to cases where alleles with contrasting effects are present in multiple loci in both parental lines. An example for that are the QTLs that we mapped for rind firmness (RF); In *RF2.1,* “Dulce” allele is associated with firmer fruit, while in *RF8.2,* “Tam Dew” allele is associated with increased firmness. These two QTLs are acting additively (no interaction) and therefore the trans-allelic combination *RF2.1_DUL_RF8.2_TAD_* is significantly firmer than all other combinations between these QTLs (Fig. 2i**,**Supplementary Fig. 4i, j).

### Candidate genes within earliness and ripening-behavior QTLs

The two QTL hubs in the current study, on chromosomes 3 (*QTL3.3*) and 8 *(QTL8.2)* provide a genetic explanation for the correlations between the different earliness and ripening-related traits. These two multi-trait QTLs are responsible for more than 30% of the genetic variation (Fig. 2c, Table 2) and are consistent with QTLs published in other studies on melon ripening behavior using different populations and genetic backgrounds [14, 18]. By breaking down earliness to its components—days to flowering and flowering to harvest— we were able to map QTLs for DtF and FtH to independent genomic loci (*DtF8.1*, *FtH3.3*, *FtH8.2*, Fig. 2a) and demonstrate independent genetic regulation of these traits. This dissection facilitates potential selection of favorable allelic combinations, possibly bypassing the negative correlation between earliness and climacterism.

QTL mapping has triggered over the last 30 years fundamental advancements in the ability to genetically dissect variation in complex traits. While this process has evolved exponentially due to NGS technologies [63], the challenge in the current post-genomic era is in translating genetic mapping information to biological and functional insights. With the availability of reference genomes and high throughput markers technologies, distilling QTLs to the candidate gene and causative polymorphism level is becoming the critical and limiting step in the process. Fine-mapping and classical positional cloning of causative genes are very labor-intensive and costly and with the genomic tools available today, this strategy is becoming less attractive and common. The focus is therefore shifting to development and implementation of effective *in silico* approaches to nominate and prioritize candidate genes within narrow QTL intervals [64], which can be targets for validation through reverse genetics approaches.

Using a multi-layered QTL annotation and prioritization pipeline (Supplementary Fig. 2**)** we identified possible candidate genes and polymorphisms. We combined detailed genotypic profile of parental genomes with functional annotations of sequence variation. Gene expression information was also included in the process. Another important layer was the validation of significant associations in two additional multi-allelic populations derived from our diverse melon collection (*GWAS180* and *HDA10*/20, Figs. 3, 4, 5). *MELO3C011432*, a WRKY transcription factor located within *QTL3.3* that showed significant association with DtH, EtE and RF (Fig. 3), was previously reported to be involved in ripening regulation in tomato [65], and to be associated with flowering time in Arabidopsis [25]. Recently it was also suggested as a possible ethylene emission candidate in melon [66]. *MELO3C011365*, transducin/WD40 repeat-like superfamily protein modulating a variety of cellular processes, such as plant hormone responses [67], showed significant association with EtE alongside additive effect in a two-gene model when paired with the recently suggested candidate in *EtE8.2*, *MELO3C024520* [14], across the *HDA10* population (R^2^ = 0.79, Fig. 4c). Gene expression results imply that *MELO3C011365* might act as negative regulator as high expression is correlated with low EtE across RILs population segregating for climacteric ripening (Fig. 4d, e). Two interesting fruit firmness candidate genes are *MELO3C024502* in *RF8.2* and *MELO3C011553* in *RF3.1* (Supplementary Table 2). *MELO3C024502* is a beta-galactosidase involved in the degradation of hemicellulose of plant cell walls [68]. This gene is highly expressed in fruit rind, with peak at 15–36 days after anthesis (Supplementary Fig. 9) and the favorable allele in our population is associated with increase in RF by ~1 KgF cm^2^ (*R^2^ = 0.10*, Table 2). *MELO3C011553* is an increased salt tolerance 1-like (IST1) protein involved in degradative sorting mechanism of plasma membrane proteins [69], that can ultimately affect cell turgor. This gene is highly expressed in ripe fruit (Supplementary Fig. 10) and the favorable allele is associated with increased RF by ~1 KgF cm^2^ in our population (*R^2^ = 0.10*, Table 2). Both genes are also significantly associated with fruit firmness across our multi-allelic *HDA20* population (Supplementary Fig. 9b and 10b).

### Structural variation based on comparison of parental de-novo assemblies

With increasing number of *de novo* assembled genomes in model and crop plants, it is becoming apparent that structural variation is an important layer in the definition of the overall genetic variation [47]. In the current study, using cost-effective combination of short and long-read sequencing, we assembled the genomes of the two parental lines of the RILs population. We found chromosome length differences between “Tam Dew” and “Dulce” that can be accounted by rearrangements detected through the whole genome alignment, e.g. half of the 6 Mb difference between Dulce’s chromosome 3 and ‘Tam Dew’s chromosome 8 are described by large translocations detected between these chromosomes (Fig. 6d and Supplementary Fig. 7). The substantial intra-chromosomal rearrangements spanning nearly half of chromosome 6 that differ between our parents also appears in the recently published *de-novo* assemblies of “Payzawat” and ‘Harukei-3′ genomes [51, 52]. Previous SVs reported in melon, mainly attributed to transposable elements and some to meiotic crossovers [32, 33, 49, 50], but these studies were focused on events of relatively small DNA fragments (<0.5 Mb). Examples for large-scale rearrangements have been reported in barley, including two frequent large inversions (>5 Mb) found in elite barley lines that are attributed to mutation breeding and the expansion of geographical range [70]. In wheat up to 1 Mb InDels caused by *gypsy* LTR retrotransposon have been identified and attributed to unequal intra-strand recombination or double-strand break events [71]. The large SVs reported here are probably the product of several separate events, but the underlying mechanism or impact are yet to be elucidated.

### Structural variations in QTL intervals and intragenic space

In the current study, we found several structural variants between the parental genomes that are within QTL intervals. One such example is in the interval of *RF3.1*, reflected initially on the linkage map, as a rearrangement of the genetic markers. For example, based on the reference genome SNP S03_18745187 is expected to be located on chromosome 3 between 18 and 19 Mb. Instead, it is located upstream on this chromosome, between S03_142528996 and S03_14691746. Another example in this block is SNP S10_11348114, originating from chromosome 10 (Supplementary Fig. 11a). These genetic differences were confirmed as structural variation through the whole genome alignment between our “Tam Dew” *de-novo* assembly and the reference genome (Supplementary Fig. 11b). We offer two examples for SVs detected within candidate genes, both in intronic regions. The first is in *MELO3C007661*, candidate in the *DtF8.2* QTL. We found a 469 bp InDel between exons 5 and 7 in this gene (Supplementary Fig. 12c, d), allegedly encompassing exon 6 (based on the CM4.0 annotation). We validated the deletion through PCR analyses of genomic DNA of both “Tam Dew” and “Dulce” (Supplementary Fig. 12a and b) and found that this InDel is present in seven additional lines from our core collection (Supplementary Fig. 12e). However, through cDNA sequencing, we show that exon 6 in the CM4.0 gene model is most likely an annotation artefact as it is absent in mRNA of both parents (Supplementary Fig. 12c). We suggest an alternative gene model based on these results, which is also supported by the “Harukei-3” CDS (Supplementary Fig. 12d, [52]). The second example is in *MELO3C004349*, a serine/threonine-protein kinase within *FF5.1* QTL interval. In this case, the SV analysis identified a 4 Kb repeat contraction in “Dulce”, between exons 1 and 2, in a region encompassing an LTR/Copia transposable element present in both the reference genome and “Tam Dew” (Supplementary Fig. 13a, b). The result is a gene model shorter by 4 Kb in “Dulce” (Supplementary Fig. 13c). In both cases we provide adjusted gene models for our parental genomes, though it is unclear what, if any, is the effect of these alterations on the CDS or expression levels as shown in recent studies that connected SVs with functional variation in tomato and melon [48, 52].

Our parental genome assemblies also allowed analysis of presence-absence variation (PAV). Out of the 1412 genes missing from either “Tam Dew” or “Dulce”, none were found within a QTL interval. Nonetheless, recent publications report on PAVs related to melon domestication in a region on chromosome 5 containing resistance genes, such as the protein coding *Vat* (*Virus aphid transmission*) [49, 50]. Though “Tam Dew” and “Dulce” are both elite cultivars (*C. melo ssp. melo*), we report here a similar PAV between these lines in *Vat* proteins, as six open reading frames on chromosome 5 are present in “Tam Dew” but missing from the “Dulce” genome (Supplementary Table 6).

We believe that further examination of the genomic data generated in this study will expose additional cases of SVs within genes, some of which with potential impact on phenotypic variation. However, the lift-over process used in this work is limited to the reference transcriptome, and at times found inaccurate—e.g. the above mentioned *MELO3C007661* gene was missing from “Tam Dew” annotation, and was manually added after the PCR validation. It is possible that *ab-initio* gene annotation supported by expression data originating from each of the parents would greatly increase the confidence of both SV and gene annotation from their present draft status.

## Conclusions

Earliness and ripening behavior in melon are shown here and in other studies to be under complex genetic control [14, 16–18]. Breeding varieties with combination of negatively correlated traits such as earliness, long-shelf life and climacteric properties is a desired and challenging goal [15]. QTL mapping facilitate the dissection of these traits to discrete elements that can be used to assemble favorable genetic combinations. In the post-genomic era, where reference genomes are available for most crop plants, detailed characterization of all levels of genetic variation is feasible. The use of resequencing of diverse accessions alongside whole genome *de novo* assemblies of parental lines of a segregating population is an effective way to identify and prioritize candidate genes within QTL intervals, towards the complementary use of reverse genetic approaches (e.g CRISPR-Cas9 mediated genome editing) for breeding improved varieties.

## Materials and methods

### Plant materials and field trials

The germplasm in this study included three sets which were grown at Newe-Ya’ar Research Center, northern Israel (32°43′05.4″N 35°10′47.7″E). The first population, TAD×DUL RILs, is composed of 164 F_7_ recombinant inbred lines originating from a cross between the late non-climacteric “Tam Dew” (TAD; *C. melo* var. *inodorous*) and the early climacteric “Dulce” (DUL; *C. melo* var. *reticulatus*) growing conditions and experimental design previously described in Oren et al., 2020 [39]. Briefly – all the RILs, F_1_ and their parents were represented by five plants per plot in two replicates and grown in a randomized block design (RCBD) in the open field in the summers of 2016 and 2017. In the summer of 2018, each line was represented by five replicates of a single plant and were grown in a 50-mesh net-house in RCBD. The second population, Melo180 GWAS panel, is composed of 177 diverse accessions representing the two melon subspecies (ssp. *agrestis* and ssp. *melo*) and eleven horticultural groups. Here, each line was represented by three plots of five plants each in an RCBD in the open field in the summer of 2015 [73]. The third population, *HDA20* –multi-allelic population of 190 F1 hybrids derived from intercrossing in a half-diallele mating scheme of 20 diverse core accessions, selected to represent the genetic variation in our Melo180 GWAS panel [56]. The 190 F1 hybrids alongside their 20 parents were grown and phenotyped in the open field in the spring–summer season of 2018. Three plots of five plants each in a RCBD experiment represented each genotype. *HDA10* is a core subset of 10 parents and 45 half-diallele F1 hybrids that are included in the *HDA20* populations.

### Trait evaluation

At maturity a single fruit from each plant was harvested at maturity based on abscission in climacteric fruits, or rind color and days after fruit set (45–50 days) and rind color in non-climacteric fruits, giving a total of five mature fruits per plot (10 per genotype). In the open field, female flowers were routinely tagged at anthesis, over the course of three weeks, and the flowering date of tagged fruits was collected during harvest. Earliness (DtH) is defined as the number of days from sowing to harvest. Time to flower (DtF) is the number of days from sowing to anthesis and fruit development time (FtH) was the number of days from anthesis to harvest. In the net house, flowers were manually pollinated and due to variation in setting, DtF data from this experiment was not reliable enough, therefore only FtH data was used (Supplementary Fig. 3c). Ethylene emission measurement was done using a previously described method [55]. Briefly, each fruit was incubated at room temperature for 30 minutes in an inert vacuumed bag. A sample of 1 ml was taken from each bag using a hypodermic syringe and analyzed in a gas chromatograph (HP 5890 Series II PLUS GC with FID; Hewlett-Packard, Palo Alto, CA, USA) equipped with an SS-packed HAYESEP Q column (80/100, 60 9 1/8″; Restek, http://www.restek.com/). Ethylene emission rate (EtE)—μL Kg^−1^ fresh weight per hour—was calculated from the sample peak area based on the standard peak area (1 ml of 1 ppm ethylene in N_2_). Fruits were then cut along the longitudinal section, and firmness — KgF cm^−2^ —was measured on each fruit at two opposite points in both flesh and rind, using a digital force gauge (M5–50 with a 12.7 mm cone point – G1026; Mark-10, Copiague, NY, USA). Fruit rind and flesh firmness (RF, FF) scores were an average of the two sampling points. Flesh sugar content, evaluated as total soluble solids (TSS) was measured by refractometer (Atago PAL-1, Atago, Japan) in juice squeezed from five fruits per plot. Genotype least square means for EtE, RF, FF and TSS were calculated on a minimum of four fruits per genotype.

### Statistical analyses

JMP ver. 14.1 statistical package (SAS Institute, Cary, NC, USA) was used for statistical analyses as described in Oren et al., 2020 [39]. Briefly, after confirming homogeneity of variances and normal distribution of traits a factorial mixed model (REML) was used for the analysis of variance, with RILs and blocks as random effects. Narrow-sense heritability (*h*^2^) was estimated for each trait in each year separately using ANOVA based variance components [73]. Trait correlations across years were calculated from least square genotype means (LS Means).

### DNA preparation, genotyping, and map construction

Extraction of DNA was done using the GenElute™ Plant Genomic Miniprep Kit (Sigma-Aldrich, St. Louis, MO), and the quantity and quality was determined using Nanodrop spectrophotometer ND-1000 (Nanodrop Technologies, Wilmington, DE), electrophoresis on agarose gel (1.0%) and Qubit® dsDNA BR Assay Kit (Life Technologies, Eugene, OR).

Genotyping of the TAD×DUL RILs was based on GBS, and map construction were previously described by Oren *et al.* [39]_._ Map construction was based on 89 343 SNPs across 146 lines. SNP filtration were done with TASSEL v.5.2.43 [74] and linkage maps construction was done using the ASMap R package [75]. Genotyping of the GWAS180 diversity panel was performed using GBS, as described by Gur *et al.* [72] and the final SNP set included 23 931 informative SNPs across 177 accessions. DNA of the founder lines of the *HDA20* population was extracted and shipped to the Genomic Diversity Facility at Cornell University (Ithaca, NY) for WGS to an estimated 30× coverage, yielding 4 million informative SNPs as previously described [57].

### RNA isolation, sequencing and differential gene expression analysis

For expression analysis, fruit rind tissue was sampled into two biological replicates from “Tam Dew” and “Dulce” at flowering day, 5, 10, 15 and 20 days after anthesis (DAA) and at mature stage. Each biological replication consisted of bulked tissue from three fruits sampled from different plants from each line. Fruit tissue was frozen in liquid nitrogen and stored in −80°C. Total RNA was extracted from 24 tissue samples (two genotypes × six developmental stages × two biological replicates) as previously described [55] and 50 μg RNA from each sample was used to construct strand specific RNAseq libraries, using Verso cDNA kit (Thermo Fisher Scientific, Grand Island, NY, USA) according to manufacturer’s protocol. Twenty-four libraries were sequenced on illumina HiSeq 2500 platform at Technion facility and yielded an average of 18 million reads per library. RNAseq analysis methods are detailed in Galpaz *et al.* [55] . In essence, trimmed and filtered reads were aligned to the latest melon reference transcriptome (CM4.0, v3.6.1 [33]) and for each melon gene raw counts were used to calculate FPKM values for 29 364 genes.

### High molecular weight (HMW) DNA extraction

A modified CTAB protocol based on Fulton *et al*. [76] was used on three weeks old seedlings etiolated for 48 hours. Approximately 1gr of fresh tissue was snap frozen and grounded with a mortar and pestle instead of a drill. Wide bore tips were used for pipetting and all mixing and inverting was done gently, without vortexing.

### Long-read DNA sequencing

High-quality HMW DNA libraries for Oxford Nanopore MinION were constructed and DNA size selection was performed using BluePippin system (Sage Science, Inc.). Library preparation was performed with 1–1.7 μg DNA using the Ligation Sequencing Kit SQK-LSK109 (ONT, Oxford Nanopore Technologies) following manufacturer’s guidelines. Libraries were loaded on MinION FLO-MIN106D flow cell. Base calling was done using the GPU version of Guppy v2.1. “Dulce” samples produced 1.7 million sequences with a sum length of 23.3 Gb between 70 bp and– 148 592 bp with an average length of 13 729 bp. “Tam Dew” produced 1.7 million sequences with a sum length of 15.7 Gb between 76 bp and 117 396 bp with an average length of 117 396 bp. Mean read qualities for both samples were equal or above Q10.

### Genome assembly

*De-novo* assemblies and their annotations were created for both parental lines of the TAD×DUL RILs. The assembly workflow is described in Supplementary Fig. 1–prior to assembly, adapter removal from ONT long-reads was performed with Porechop [77] using default parameters. Assembly was performed using the Flye assembler [78], genome size set to 400 Mb and coverage was set to 50 for Dulce and 35 for “Tam Dew”. Default values were used for all other parameters. Each set of contigs was polished with Racon in three rounds (v1.4.7,) [79] using default parameter settings, followed by three rounds of polishing using Pilon (v1.23,) [80] with the illumina paired-end reads after tagging duplicate artifacts using Picard MarkDuplicates (“Picard Toolkit.” 2019. Broad Institute, GitHub Repository. http://broadinstitute.github.io/picard/; Broad Institute). Both long and short-reads were aligned using Minimap2 (v2.17) [81] with parameters set to default values. Sorting and conversion of mapping files were performed with SAMtools [82]. Polished contigs were scaffolded according to the Melon v4.0 reference genome [32] using RaGOO (v1.1) [83]. Assembly stats and evaluation were produced using seqkit (stats -a -G N, [84] and QUAST (v5.0, —large) [85]. Finally, BUSCO was used to assess genome completeness (v4.1.2) [86].

### Repeat analysis and gene annotation

RepeatModeler2 [58] was used with -LTRStruct to characterize *de-novo* repetitive elements in both “Dulce” and “Tam Dew” genomes. Gene models were annotated using a lift-over approach based on the Melon v4.0 data previously published [32, 33]. We initially used GEAN [60] based on the reference melon coding sequences (CDS), following best practices as detailed in the manual. We later complemented the results with Liftoff (−exclude_partial -a 0.95 -s 0.95) [59] using default parameter settings, later filtering out results with sequence identity less than 90%.

### Structural variation analysis

To observe SV variation between “Dulce”, “Tam Dew” and Melon genome V4.0, we first aligned the assemblies to each other and to the reference using Nucmer (v3.1 [87], −maxmatch -l 100 -c 500). We then used Assemblytics [62] (unique_length_required = 10 000 min_size = 50, max_size = 100 000). Additional annotations of inversions or translocations was added to SV’s detected based on orientation and location. These results were then compared to syntenic dotplots generated using Synmap2 on the CoGe platform using default values [88, 89].

### Variant annotation and protein alignments

Variant annotation and effect prediction of the VCF from the WGS of the 25 core accessions, were carried out using SnpEff with default parameters [54], based on the latest version of the melon genome fasta sequence and gene models (Melon_v4.0) [32] to construct a melon SnpEff database. In parallel, amino acid substitution effects were also categorized as tolerant or non-tolerant (radical) using SIFT [90] and PROVEAN [91]. Orthologue proteins were blasted using NCBI’s nr database, within dicotyledonae, using default parameters, and the view was generated using NCBI’s Multiple Sequence Alignment Viewer (ver. 1.19.2).

### QTL analysis

QTLs were analyzed as previously described [39]. In brief, TASSEL ver. 5.2.51 [74] was used for genome-wide linkage analysis of the traits using a generalized linear model (GLM) with 1000 permutations and a *p-value* of 0.05 as threshold. Interval mapping, both standard and stepwise, were performed with R/qtl (v1.44, Broman et al. 2003), with 1000 permutations and *p-value* of 0.05 as detection threshold using 1.5 LOD scores confidence intervals. Composite interval mapping (CIM) was done based on a 10 cM marker window size.

### Scoring of candidate genes within QTL intervals

To classify and rank polymorphisms within predicted genes, we used SnpEff [54] that predicts and classifies the effect of variants on annotated genes. We start by scoring the genes proximity to the QTL peak (<LOD 0.5 + 2, between LOD 0.5 and LOD 1.0 + 1, >LOD 1.5 + 0). If a gene within the QTL interval contains a non-synonymous polymorphism, then its score is weighted based on the impact of that polymorphism as classified by SnpEff (modifier +0.5, low +1.0, moderate +1.5, high +2.0). After examining the genes’ description, excluding unknown or non-relevant annotations, we follow up with available data for spatial and temporal expression data, once again, adding a score for the relevant results (as described in the flowchart, Supplementary Fig. 2). The score matrix is then translated to a “general” score, between 1–10, for each gene.

## Acknowledgments

We wish to thank Uzi Saar, Fabian Baumkoler and the Newe-Yaar farm team for technical assistance in setting the field trials and for plant maintenance. We thank Dr. Vitaly Portnoy for technical assistance in ethylene emission measurements, and Dr. Nurit Katzir for sharing raw data on the PI414xDUL RILs population. Funding for this research was provided by the United States-Israel Binational Agricultural Research and Development Fund (BARD) grant no. IS-4911-16 and by the Israeli Ministry of Agriculture Chief Scientist grants no. 20-01-0141.

## Author contributions

EO and AG conceived the research plan and designed the experiments. YB, AD and AG were responsible for development of plant genetic materials. EO, AD, GT, TI and AG performed the experiments and collected the data. ERR, BS and ESB contributed to the genomic sequencing and *de novo* assembly pipeline. Bioinformatic, genomic and statistical support were provided by YE, SF, YT and AAS. EO analyzed the results. EO and AG wrote the manuscript. All authors discussed the results and approved the final version of the manuscript.

## Data availability statement

The data supporting the findings of this study are available within the paper and within its supplementary materials published online. Raw sequences and FASTA files of genome assemblies can be found at NCBI BioProject: PRJNA775626.

## Conflict of interest

The authors declare that they have no conflict of interest.

## Supplementary data

Supplementary data is available *at Horticulture Research Journal* online.

## Supplementary Material

Web_Material_uhab081
