# Supplementary material for: QTL mapping and genomic analyses of earliness and fruit ripening traits in a melon recombinant inbred lines population supported by de novo assembly of their parental genomes
Source: Hortic Res. 2022 Jan 19;9:uhab081. doi: 10.1093/hr/uhab081 (PMC8968493; doi:10.1093/hr/uhab081)
Supplement: Web_Material_uhab081 [file web_material_uhab081.zip › Supplementary Figures.pdf]

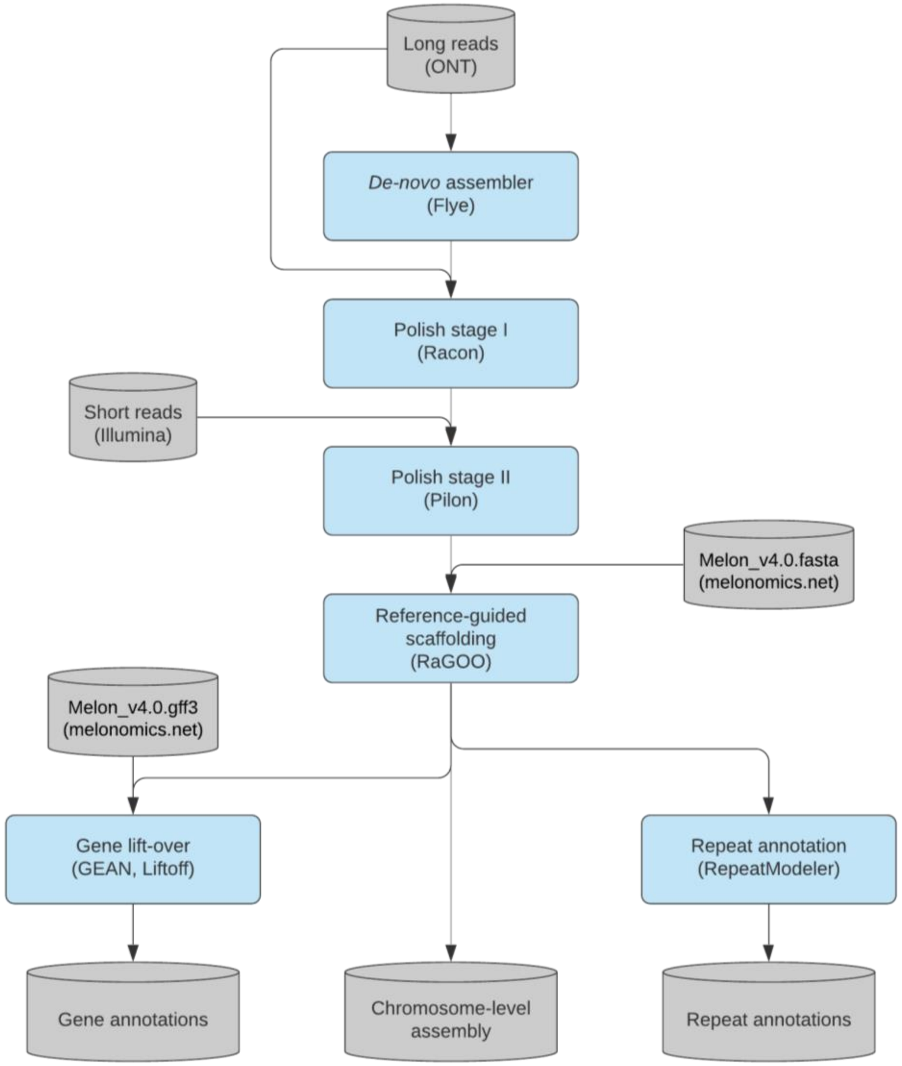


**Supplementary figure 1:** *De-novo* assembly workflow

**Supplementary figure 2:** Workflow for QTL annotation and prioritization of candidate genes

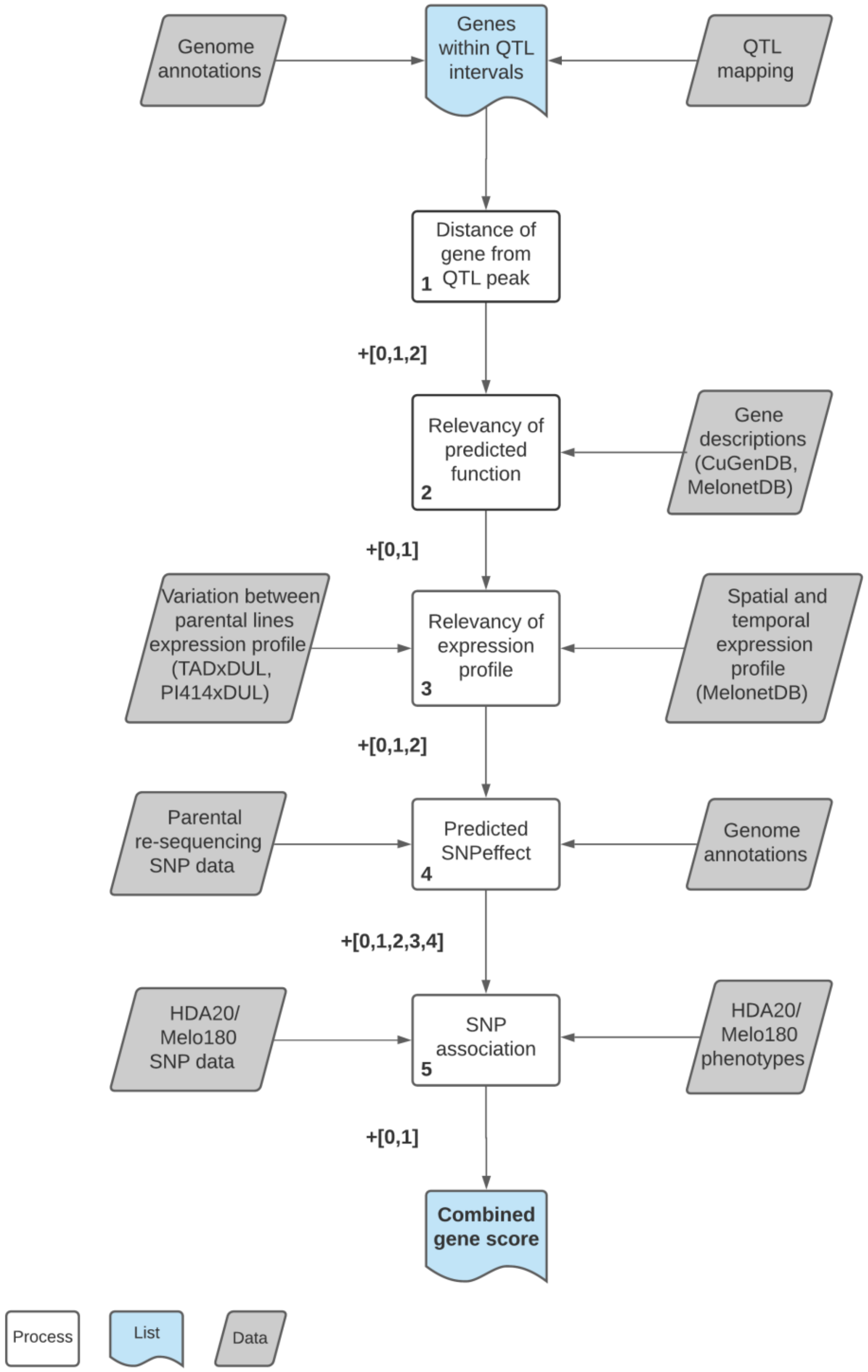

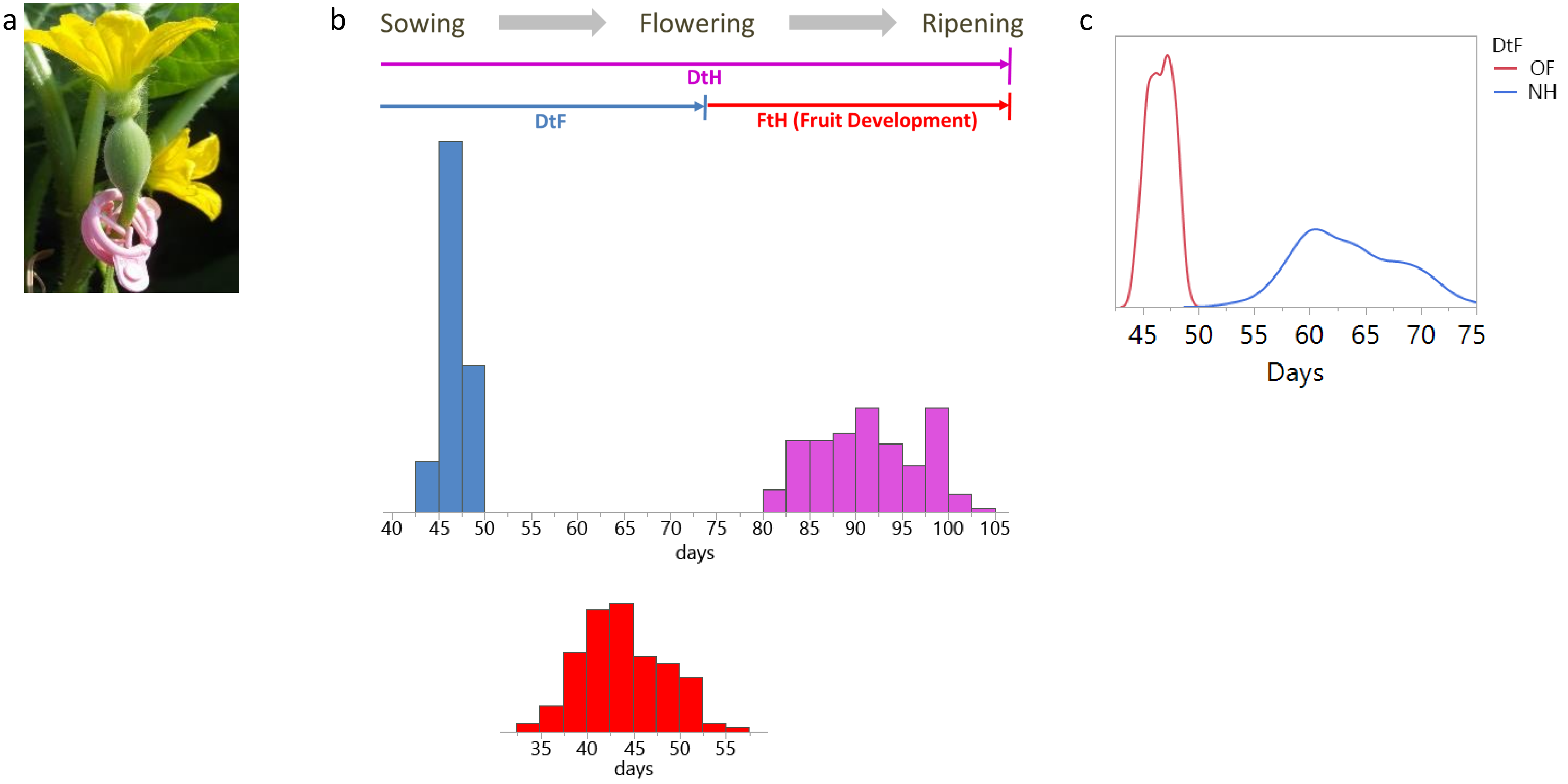


**Supplementary figure 3:** Dissection of earliness (days from sowing to harvest) in Tamdew x Dulce RILs. a) female flower tagging at time of anthesis. b) Distributions of the earliness and its components in different color along days axis. Days to harvest (DtH) in purple, days to flower (DtF) in  blue, fruit development time (FtH) in red. c) DtF distribution in open field (red) with  bee pollination and net house (blue) with manual pollination.

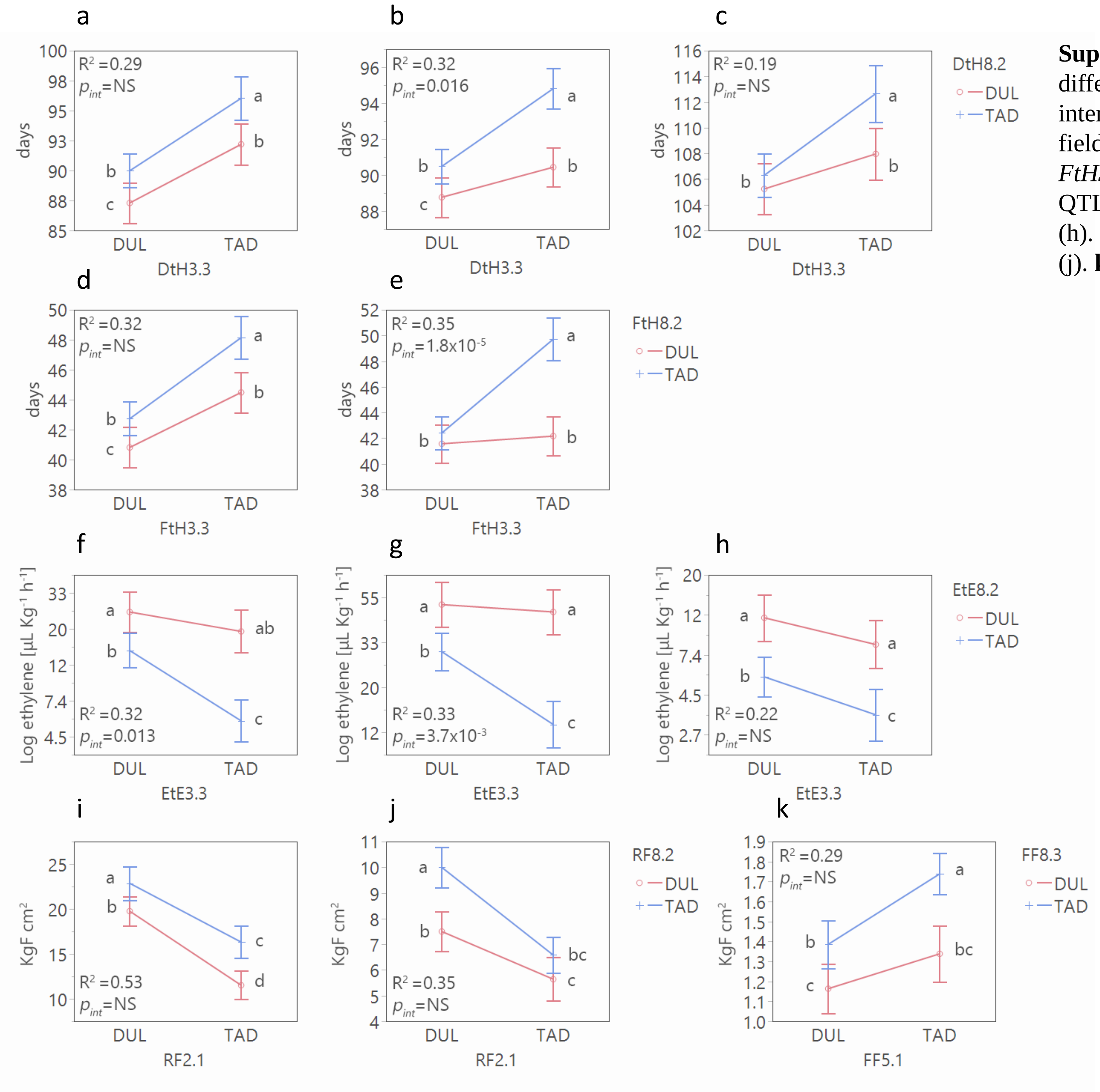


**Supplementary figure 4:** QTL Interaction plots in the TADxDUL RILs. Statistically different means designated by different letters. $P_{int}$ represent significance of the interaction between QTLs. **a-c**) Interaction of QTLs *DtH3.3* and *DtH8.2* in the open field – 2016 (a). Open field- 2017 (b). Net house-2018 (c). **d-e**) Interaction of QTLs *FtH3.3* and *FtH8.2*: Open field-2016 (d). Net house-2018 (e). **f-h**) Interaction of QTLs *EtE3.3* and *EtE8.2*: open field-2016 (f). Open field-2017 (g). Net house-2018 (h). **i-j**) Interaction of QTLs *RF2.1* and *RF8.2*: Open field-2017 (i). Net house-2018 (j). **k**) Interaction of QTLs *FF5.1* and *FF8.3*, Net house-2018 (k).

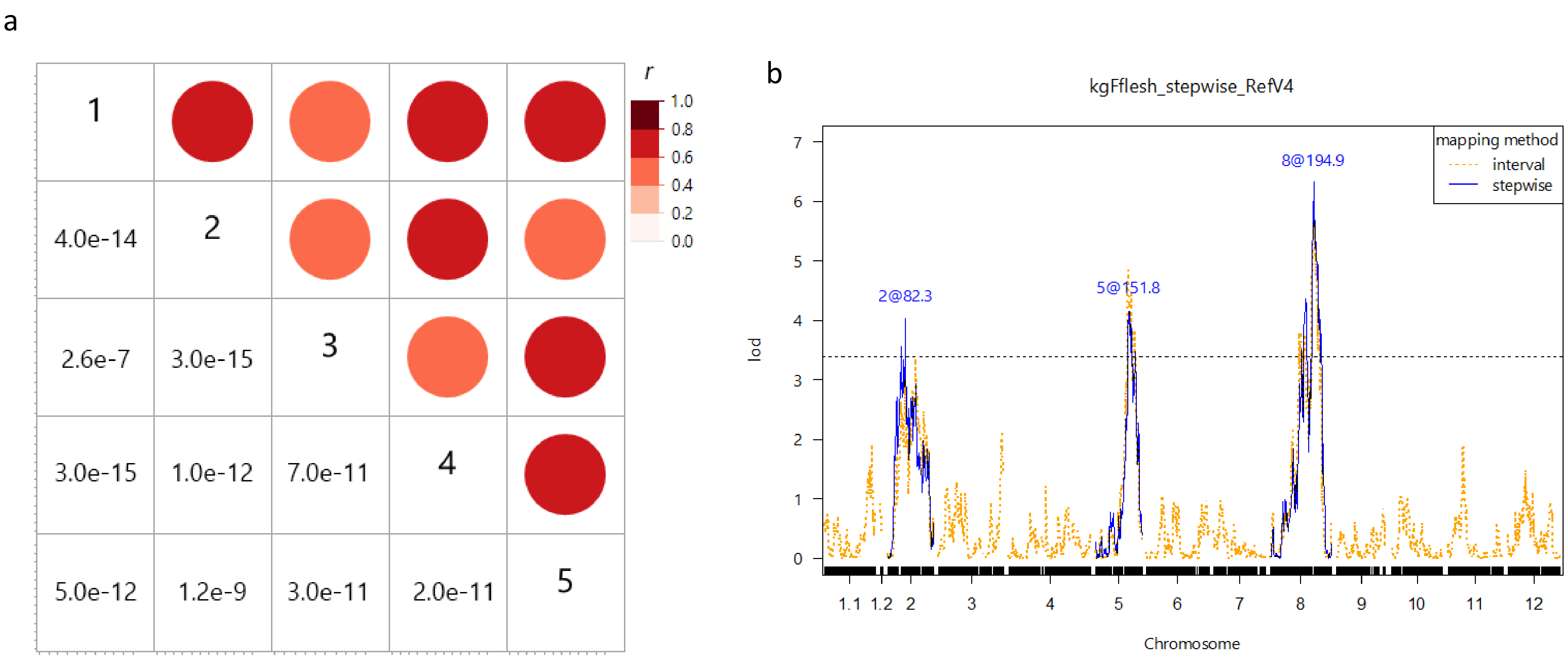


**Supplementary figure 5: Analysis of Flesh Firmness (FF) in the TADxDUL RILs. a**) correlation between five replicates of the TADxDUL RILs population with their respective p-values in the lower left side b) FF genome wide QTL plot based on interval (orange) and stepwise (blue) analyses.

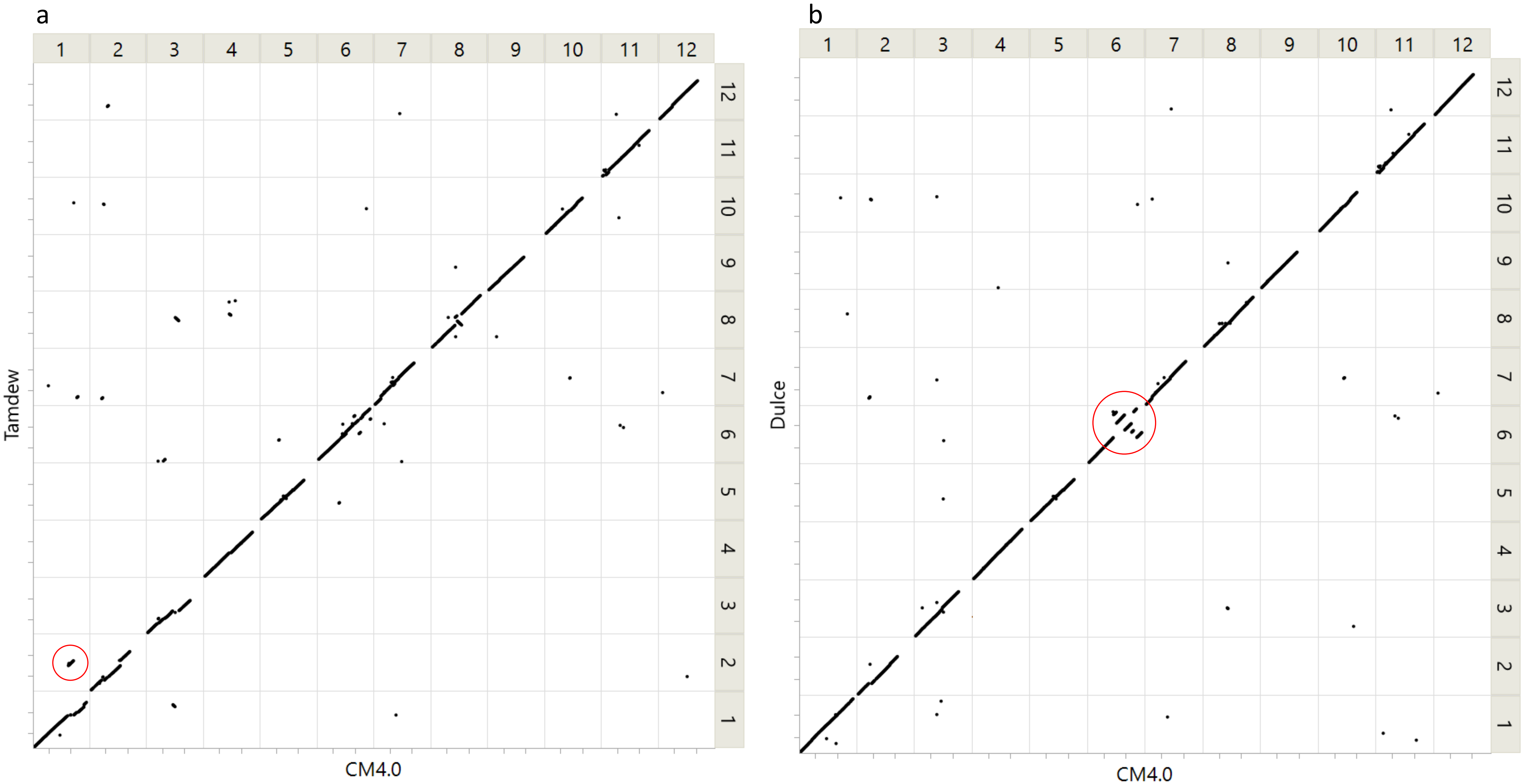


**Supplementary Figure 6: Genomic alignment between CM4.0 and the parental genomes.** **a**) Tamdew versus CM4.0 **b**) Dulce versus CM4.0. Large structural variation examples mentioned in the text are circled in red.

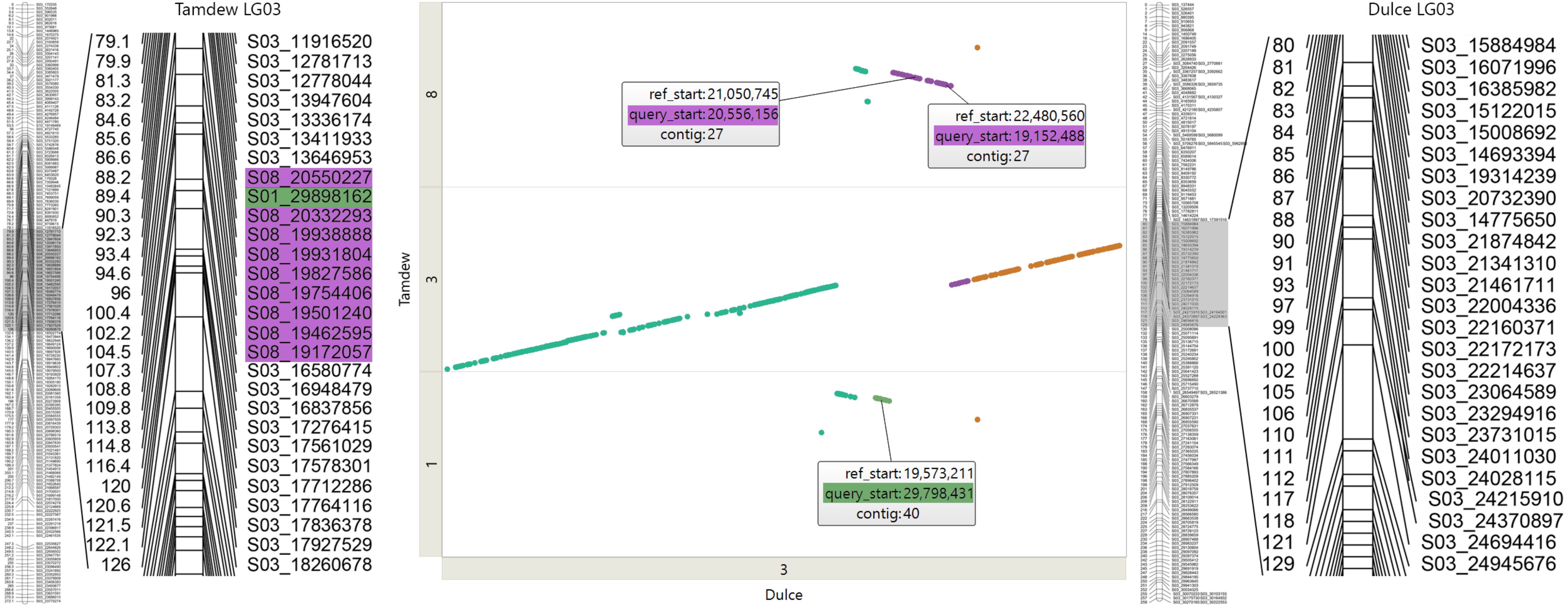


**Supplementary Figure 7:** Example for SVs detected through whole genome alignment between Dulce's chromosome 3 and Tamdew's chromosomes 1 and 8. These genomic rearrangements were confirmed through linkage maps generated separately for each genome.

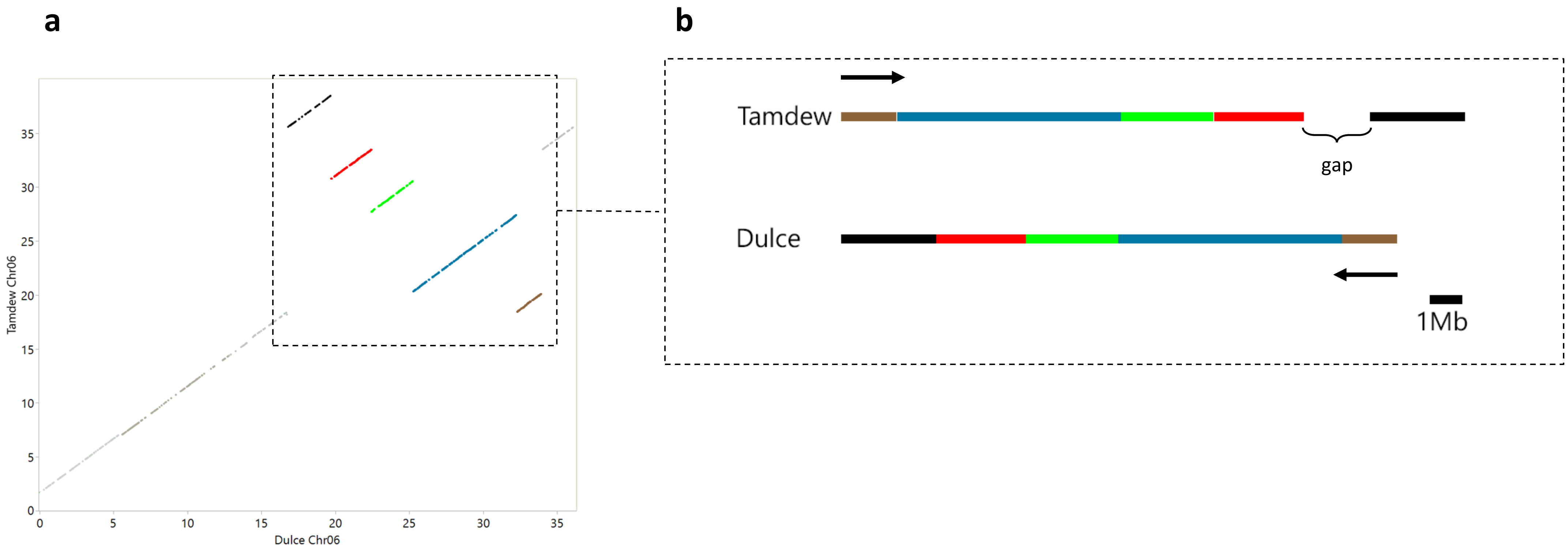


**Supplementary Figure 8: Chromosomal rearrangement between Tamdew and Dulce on chromosome 6**. a) whole chromosome alignment view. b) illustration of the de novo assemblyies of Tamdew and Dulce in this region.

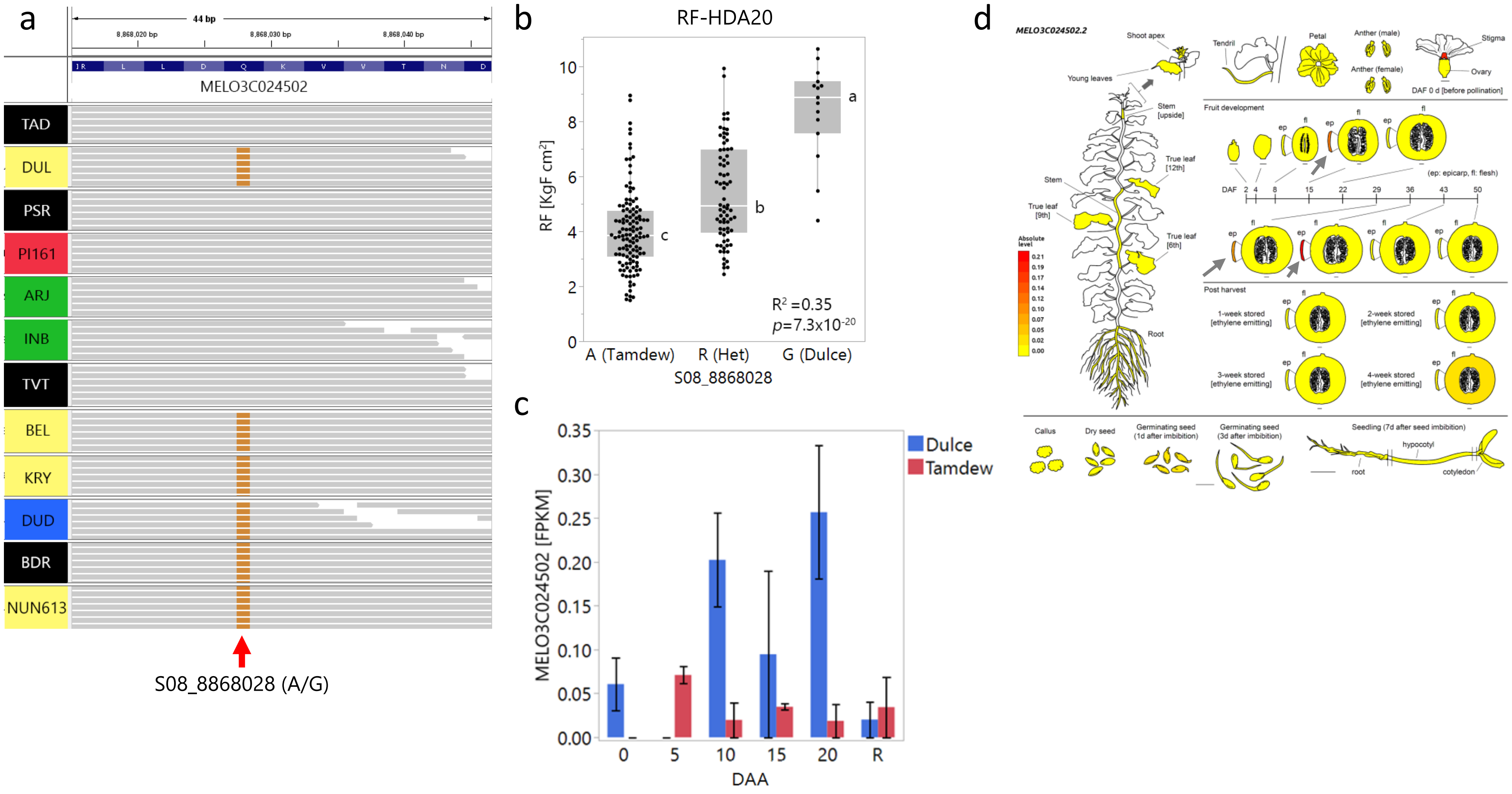


**Supplementary Figure 9: Characterization of MELO3C024502-Beta-galactosidase gene**. **a**) SNP S08_8868028 in MELO3C024502 across representative core accessions, including the parental lines Tamdew and Dulce. Color coded according to horticultural group. **b**) Association of SNP S08_8868028 with Rind Firmness (RF) in *HDA20* population. **c**) Expression profile of MELO3C0240502 from Tamdew and Dulce rind samples through fruit development. **d**) Spatial expression profile of MELO3C024502 as presented in MelonetDB (Yano et al. 2018). Arrows mark tissues with high expression levels.

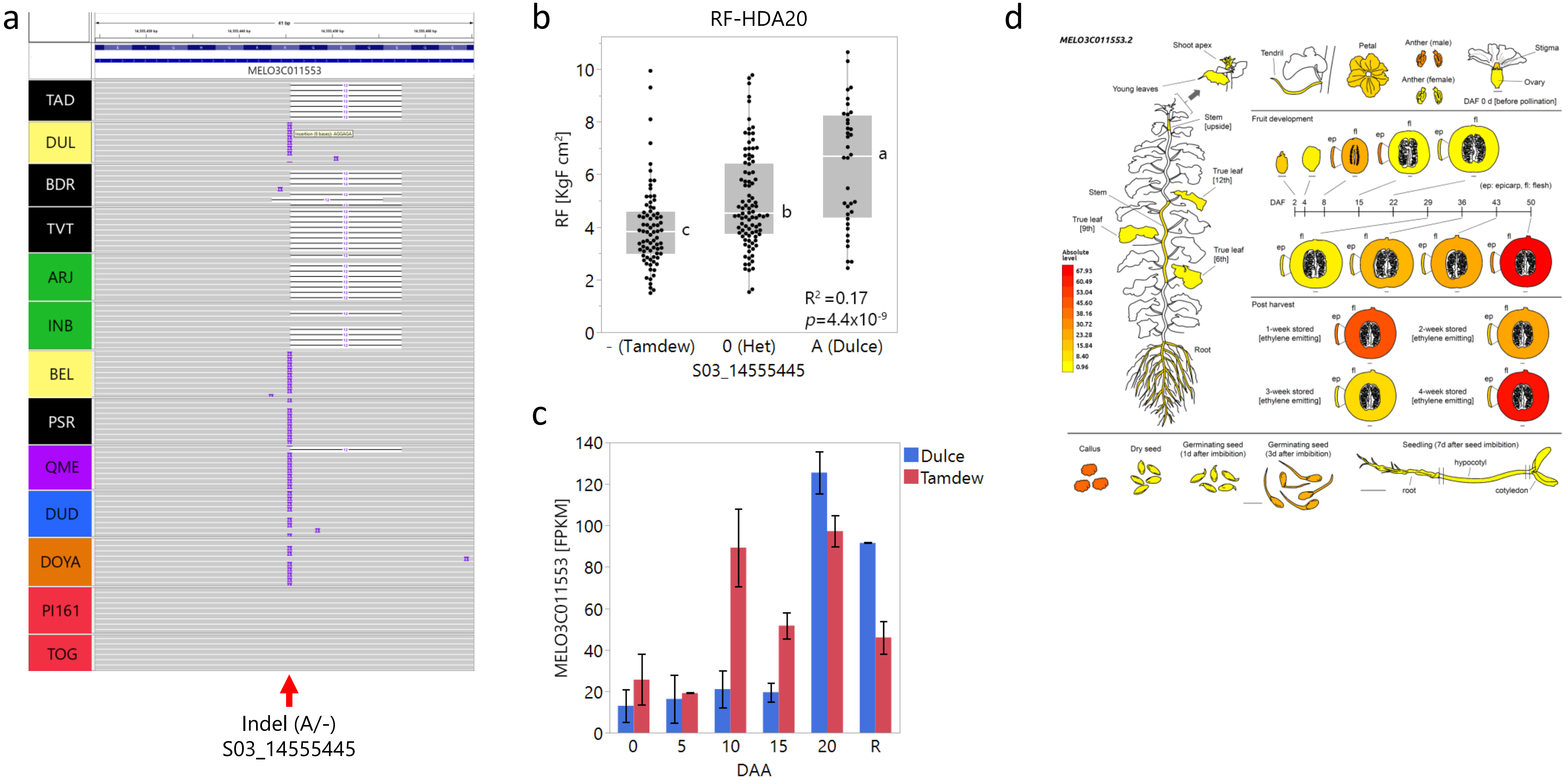


**Supplementary Figure 10: Characterization of InDel in MELO3C011553-IST1-like protein. a**) 6bp InDel in MELO3C011553 across representative core accessions, including the parental lines Tamdew and Dulce. Color coded according to horticultural group. **b**) Association of InDel in MELO3C011553 with Rind Firmness (RF) in HDA20. c) Expression profile of MELO3C011553 from Tamdew and Dulce rind samples through fruit development. d) Spatial expression profile of MELO3C011553 as presented in MelonetDB (Yano et al. 2018).

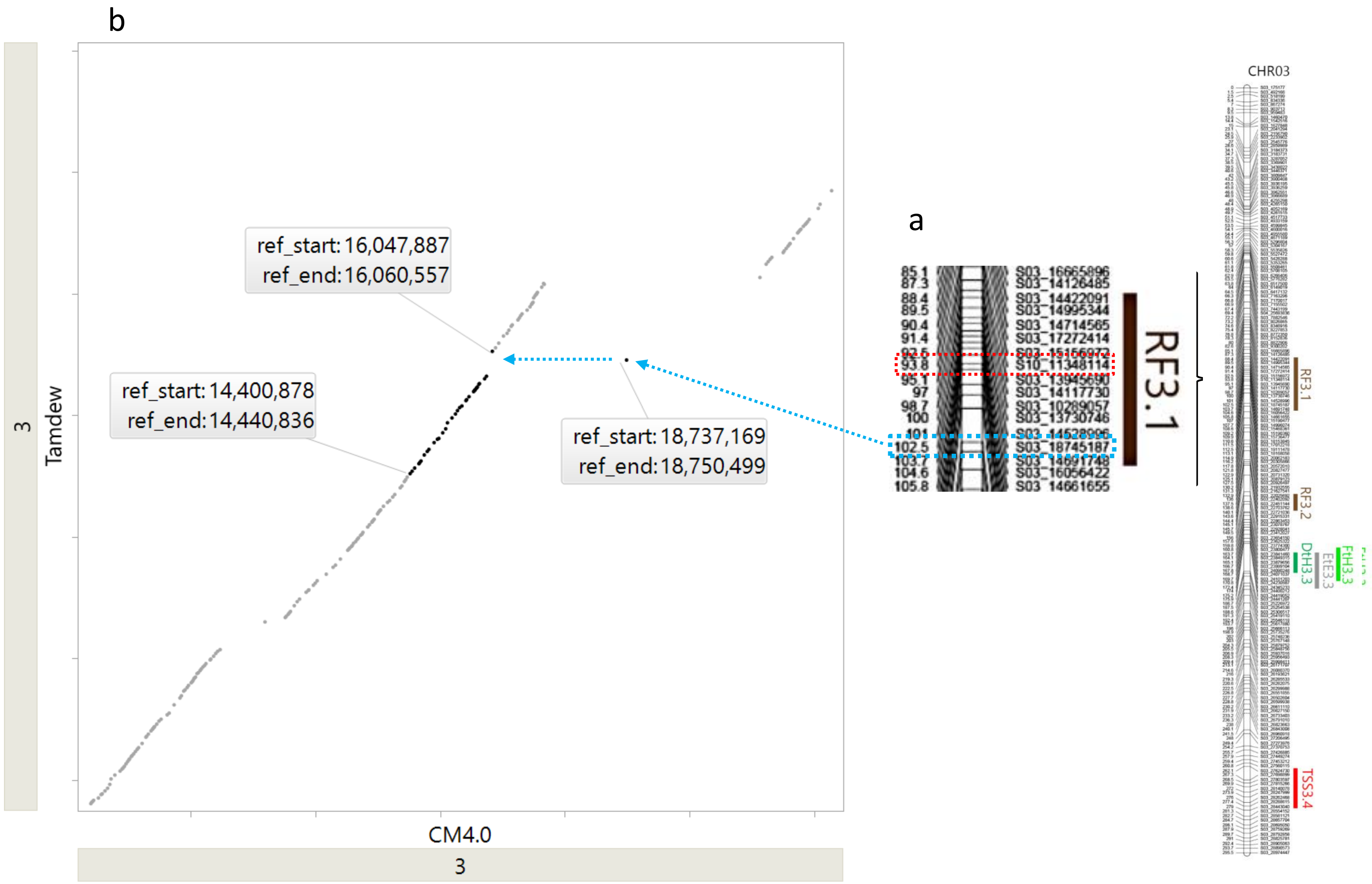


**Supplementary figure 11:** Genomic rearrangements within *RF3.1* physical interval. **a**) An example of a local rearrangement as detected through whole genome alignment. **b**) An example for trans (different chromosome- chr10) and cis (same chromosome) translocations as detected through linkage analysis on the TADxDUL RILs population.

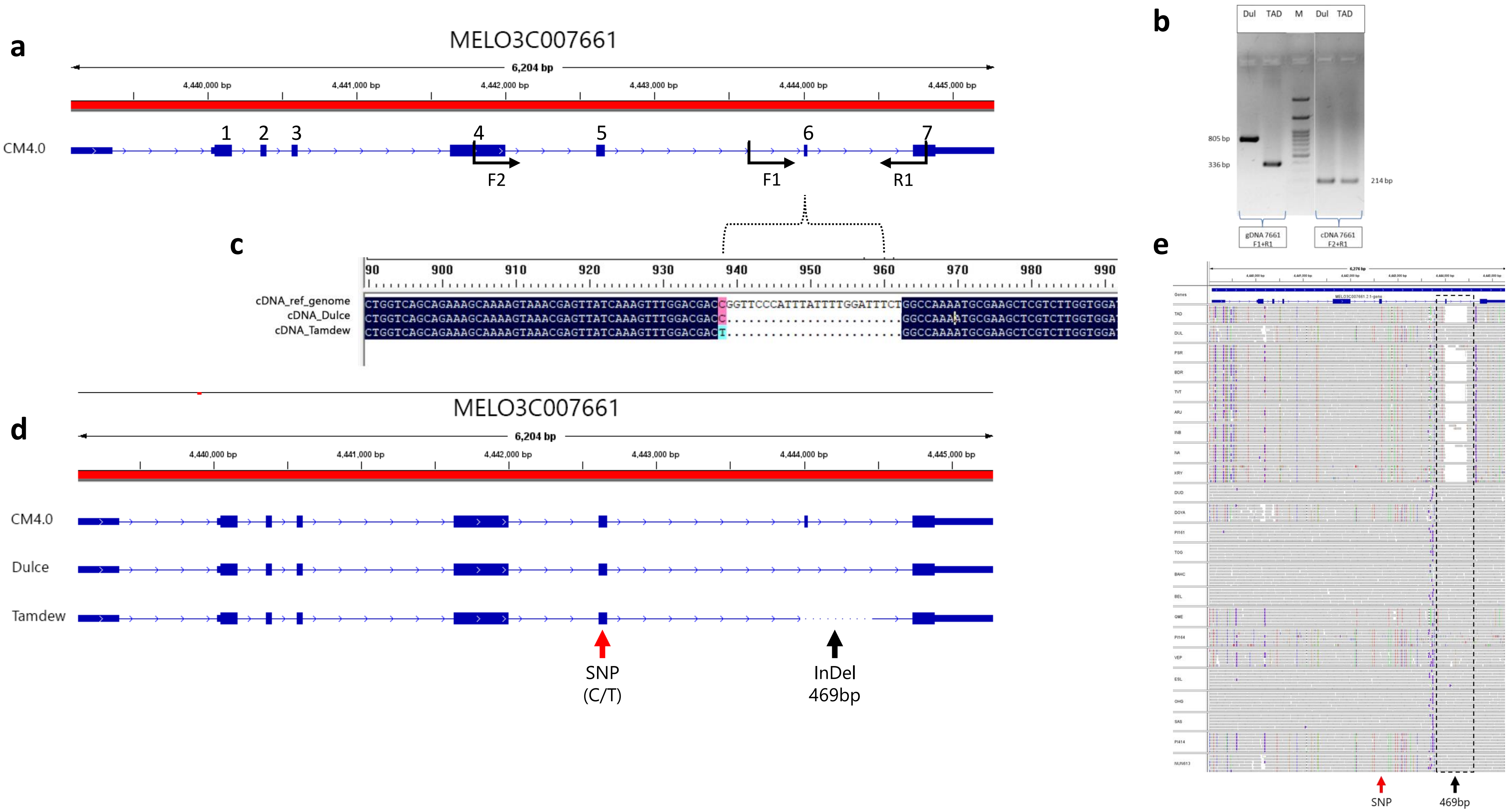


**Supplementary figure 12: Structural variation in MELO3C007661 a**) MELO3C007661 gene model and primers positions. **b**) PCR products of Tamdew and Dulce gDNA and cDNA. **c**) Sanger sequencing results of Dulce and Tamdew cDNA, aligned to Cm4.0 gene transcript. Exon 6 is missing from both Tamdew and Dulce. SNP in exon 5 (cCg/cTg) marked in red and blue. d) Gene models for CM4.0, Dulce and Tamdew. Exon 6 is missing from both Tamdew and Dulce. Black arrow marks region where the 469bp InDel was detected in the Tamdew *de novo* genome. Red arrow marks the reported SNP in exon 5. e) 469bp deletion within MELO3C07661**.** A comparison of the 25 lines WGS bam files show the deletion, marked by the black arrow, in Tamdew and seven additional lines.

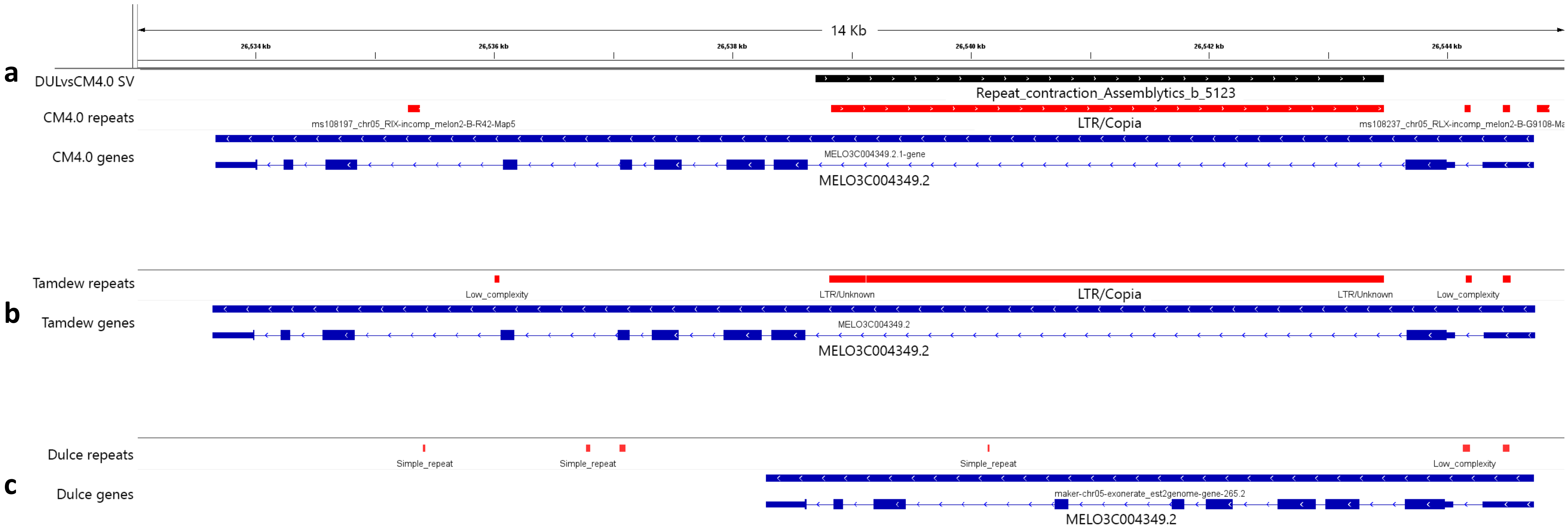


**Supplementary Figure 13: Structural variation between exons 1 and 2 in MELO3C004349.** Repeat contraction between exons 1 and 2 changed gene model in Dulce which is 4K shorter. Black - result of the SV analysis between Dulce and CM4.0. Red - repeat annotation of LTR/Copia element in are in red. Blue - gene models. a) CM4.0 annotations. b) Tamdew annotations. c) Dulce annotations.
